# Experiences of Pacific Peoples and their ‘āiga/kāinga/kāiga/vuvale living with Parkinsons: A qualitative study

**DOI:** 10.1371/journal.pone.0355989

**Published:** 2026-08-12

**Authors:** Charleen Silcock, Megan Lupe, Salote Makasini, Leigh Hale, Christopher Higgs, Rose Richards

**Affiliations:** 1 Centre for Health, Activity and Rehabilitation Research, School of Physiotherapy, University of Otago, Dunedin, New Zealand; 2 Va’a o Tautai – Centre for Pacific Health, University of Otago, Dunedin, New Zealand; University of North Texas Health Science Center, UNITED STATES OF AMERICA

## Abstract

Parkinson’s is one of the fastest growing neurological disorders worldwide, yet little is known about this disorder in Pacific Peoples in Aotearoa New Zealand. This study aimed to explore the experiences and impact of Parkinson’s on Paciﬁc Peoples living with Parkinson’s and their ‘āiga/kāinga/kāiga/vuvale [family] in Aotearoa New Zealand. Underpinned by Talanoa research methodology [a Pacific phenomenological methodology founded on oral traditions of producing, sharing and transferring knowledge through conversation], eight consenting Pacific adults (>18 years) living in Aotearoa and diagnosed with Parkinson’s, irrespective of type, were invited to participate in talanoa [face-to-face conversations that understand the cultural relationality and connectedness of those involved]. Data were transcribed and analysed using Reflexive Thematic Analysis. Pacific researchers lead the research, data collection and analysis, and facilitated subsequent talanoa with participants and their wider communities to discuss how findings could be used to benefit these communities. The key finding was an overarching theme portraying a metaphor of a journey that participants had and were navigating from the time of their Parkinson’s diagnosis. This journey was described as travelling in unpredictable and turbulent seas in a va’a/vaka (a traditional Polynesian outrigger canoe). This overarching metaphor comprised four themes (1) An unexpected journey, (2) Who’s on the va’a/vaka with me? (3) Navigating your va’a/vaka – looking up to the stars, and (4) Steering your va’a/vaka – finding your way. For our participants, Parkinson’s was seen as a “new” health condition for Pacific Peoples. Strengthening knowledge and understanding of this disorder in ways that are acceptable and accessible to families and their communities is essential to harness the community spirit that defines Pacific culture. Healthcare services also need to improve how they offer accessible care and support Pacific communities in culturally appropriate and safe ways that considers the permanent and progressive nature of Parkinson’s.

## Introduction

Parkinson’s is a progressive neurodegenerative disorder that presents with both motor and non-motor symptoms [1]. It is one of the fastest-growing neurological disorders in the world, with the prevalence predicted to increase from 6 million to 25 million by 2050 worldwide [2]. Most people are diagnosed with Parkinson’s aged >50 years but some can develop the condition earlier in life [3].

In Aotearoa New Zealand (Aotearoa), 1 in 500 people have Parkinson’s. The 2013 age-standardised prevalence (per 100,000 population) of Parkinson’s across ethnic groups was New Zealand European, 223; Asian, 174; Pasifika, 160; and Māori, 114 [4]. The reasons for these ethnic differences are unknown but may be due to differing exposure to determinants of Parkinson’s or to factors such as data quality and inequities in access to health care [4].

Pacific Peoples make up 8% of Aotearoa’s population and comprise over 40 diverse ethnic groups. The largest populations being Samoan (49%), Cook Island Māori (21%), Tongan (20%), Niuean (8%), Fijian (5%), Tokelauan (2%), Tuvaluan (1%) and Kiribati (0.7%) [5]. Although collectively Pacific Peoples are the youngest population group in Aotearoa (median age 22.3 years, 53% under the age of 25 years) they have the third highest prevalence for Parkinson’s (160 per 100,000) of ethnic groups with known prevalence (namely, European, Asian, Māori), and, more broadly, experience inequitable health outcomes compared with non-Māori non-Pacific people in Aotearoa [5,6].

Little published research relating to Parkinson’s in Pacific Peoples in Aotearoa exists, although there is a growing body of work investigating the biallelic p.L347P PINK1 variant (PINK1 variant) early-onset Parkinson’s disease (age of onset before 51 years) in Paciﬁc Peoples in Aotearoa [7]. A recent Aotearoa retrospective chart review of demographic and clinical factors of 27 patients with genetically confirmed PINK1 Parkinson’s disease were found to be predominantly of western Polynesian ethnicity (15 were Samoan, 8 Tongan, 3 Tokelauan and 1 was Filipino), with most developing symptoms <50 years [8].

The dearth of information relating to Parkinson’s and Pacific Peoples in Aotearoa and its apparent growing prevalence prompted our qualitative study. The aim of this study was to address the research question: What are the experiences and impact of Parkinson’s on Paciﬁc Peoples living with Parkinson’s and their ‘āiga/kāinga/kāiga/vuvale in Aotearoa? With a focus on reducing health inequities, we hope this research will inform more appropriate delivery of services for Pacific Peoples living with Parkinson’s.

As this is a qualitative study focused on the perceptions of people living with Parkinson’s we have adopted throughout their preferred use of the terminology of Parkinson’s and not Parkinson’s disease. This respects their view that it is not a disease and gives credence to this umbrella term covering a range of conditions that share similar symptoms [9,10].

## Method

### Design

Underpinned by an experiential constructionist perspective, our methodological approach was that of Reflexive Thematic Analysis [11–14] with data collected via individual Talanoa (conversations). Whilst each Pacific ethnic group has its own unique cultures and traditions, common to all is the high value placed on family, collectivism and communitarianism underpinned by spirituality, reciprocity and respect [5]. Thus, our study was guided by Talanoa research methodology to privilege Pacific customs and protocols [15,16]. This methodology is based in the oral traditions of Pacific peoples and strongly values reciprocity, respect, humility and love [17] and focuses on building relationships between the researchers and the participants [18]. “Noa” means common, ordinary, or nothing in particular. “Tala” refers to communications which are about commenting, telling, relating, or informing. Thus, talanoa simply means talking about nothing within an open framework [16]. The context in which these conversations take place must however support respect and reciprocity. Ideas are explored and discussed with differing levels of complexity in open and respectful conversations or “tala” [16]. A flexible interview framework is necessary to enable deeper knowledge and understandings to develop [16].

Mindful of the shared Pacific values of respect, reciprocity, gerontocracy, service, collective responsibility, humility, spirituality and love [19, p4], a key principle of this research was reciprocity. In having the privilege of exploring our participants’ experiences and impact of Parkinson’s on their lives, and to enable this new knowledge to inform more appropriate delivery of services for Pacific Peoples living with Parkinson’s, a key question we included was “*What would you like to see come out of this research that would be of benefit to you?*” To this end, a second stage in our study was to present our findings to our participants for verification and for further discussion as to how they wished us to use these findings.

This second stage comprised several group Talanoa. Firstly, two community presentations were held, and then two further online Talanoa later with a few participants who wished to be involved.

Whilst the Reflexive Thematic Analysis methodology [12] provides a “Western” approach to qualitative data analysis, notwithstanding reservations expressed by some Pacific researchers [20], many Pacific studies have used it, considering that its processes are appropriate and complementary [17]. This is likely due to Reflexive Thematic Analysis congruence with non-positivist ‘Big Q’ Qualitative research [21,22] that embraces constructivist and interpretive paradigms of data generation and analysis. ‘Big Q’ Qualitative approaches acknowledge researcher subjectivity, and that meaning and knowledge is partial, situated and contextual [23]. This methodological approach harmonises with commonalities in Pacific research methodologies which include philosophies conceptualised around place (land) and space (relations) [24,25].

The University of Otago Human Ethics Committee (Health) approved the study (H22/021) in accordance with the Declaration of Helsinki. All participants signed informed written consent prior to data collection. Recruitment was over the period 24 July 2023–28 February 2024. Reporting of this study was guided by the Standards for Reporting Qualitative Research [26] and the Reflexive Thematic Analysis Reporting Guidelines developed by Braun and Clarke [22]. Additional information regarding the ethical, cultural, and scientific considerations specific to inclusivity in global research is included in the Supporting Information (S1 Checklist).

### The research team

Four researchers in this study were of Pacific ethnicity (CS, Cook Islands, Scotland, Tainui, Tahiti, Niue; ML, Samoan; RR, Samoan; SM, Tongan) and two were New Zealand European (LH, CH). Four were experienced physiotherapists (CS, ML, LH, CH), one in physiotherapy for people living with Parkinsons (LH). There were a mix of experienced qualitative researchers (RR, LH, CH) and early career researchers (CS, ML, SM). Four were female and one male (CH).

### Participants and recruitment

Pacific adults (> 18 years) living in Aotearoa and diagnosed with Parkinson’s, irrespective of type, were eligible to participate. To recruit participants advertisements (in English, Samoan or Tongan) were sent to appropriate stakeholders and established networks of the research team, including Parkinson’s New Zealand, New Zealand Brain Research Institute, Tangata Atumotu Trust, Parkinson’s Canterbury, the Mātai Institute, and via public channels (e.g., TV, newsletters, notice boards) and social media (e.g., Twitter, Facebook). Those interested in volunteering contacted CS or ML who discussed the study with them and provided study information sheets and consent forms to sign (in English, Samoan or Tongan as requested). On consenting to participate, an appointment at a mutually agreeable day and time an interview was made. Congruent with Reflexive Thematic Analysis, in which generated knowledge is recognised as partial and contextual [23], we recruited to achieve theoretical sufficiency, collecting data until the depth of our understanding enabled us to build theory [22].

### Data collection

One or two researchers (ML and/or CS) visited the participants in their homes. The research was explained and signed consent to participate gained. Participants could choose whether they participated in only an interview or an interview and physical assessments. Demographic data of age, gender, and Pacific village were then recorded.

For contextual understanding of the measurable physical impact of Parkinson’s on participants, information related to their Parkinson’s (time since symptoms started and since confirmed diagnosis, medications, other health issues, falls within the past year, experiences of freezing events whilst walking in the last month, Hoehn and Yahr (HY) stage [27]) was collected. Participants then underwent the following assessments: the Movement Disorder Society Unified Parkinson’s Disease Rating Scale (MDS-UPDRS) Part III, Motor Examination [28], the Time Get Up and Go (TUG) (seconds) [29], the TUG (Dual task) (seconds) [30] and Time taken to walk 4m (seconds) [31]. ML and CS were trained in the use of these tools and permission was granted for use of the MDS-UPDRS by the International Parkinson and Movement Disorder Society.

Qualitative data were collected via talanoa using open ended questions in either English, Samoan or Tongan. Based on prior consultation, these two Pacific languages were identified as the two Pacific languages to include in this study. The talanoa were facilitated by ML and CS, with initial mentoring from a senior Pacific qualitative researcher. These talanoa occurred at a mutually agreeable venue. The research team developed the interview guide, as can be seen in Box 1. Permission was gained to audio record the talanoa. The talanoa took between one to three hours. Following the talanoa, the audio-recording was transcribed word for word by a commercial transcribing company into English. No interview was solely in Samoan or Tongan, and thus the parts that were in these languages were kept in the transcript as such and not translated into English.

**Box 1. The interview guide.**

Please tell me when you were first aware you had Parkinson’s.

What were your first symptoms and when did you first notice?

Who supported you at this time?

Did you seek medical advice and what was you experience with seeking medical advice?

What things about medical support was useful?

What things could have been improved?

Please tell me about living with Parkinson’s and what it is like for you (and your aiga).

In a perfect world what would support for Pacific Peoples living with Parkinson’s look like?

Do you have advice that might be beneficial specifically to engaging/working with Pacific Peoples living with Parkinson’s and their ‘āiga.

What would you like to see come out of this research that would be of benefit to you?

How and where would they like us to talk about/ report on our findings?

### Data analysis

The demographic and quantitative data were descriptively analysed (means, ranges). The 3-step clinical prediction tool for assessing the probability of falling was calculated based on the reporting of falling in the past year and freezing in the past month, and the person’s speed of walking 4m [32].

The qualitative data analysis was undertaken by the Pacific members of our research team (CS, RR, SM, ML) guided by the Reflexive Thematic Analysis process described by Braun and Clarke [11] which requires data immersion via multiple reading, reflective questioning and deep consideration, and insightful development [11]. Given that these researchers covered a range of Pacific ethnicities, between them they were able to translate and understand Samoan and Tongan untranslated sections of the transcripts, as well as provide cultural insights. To gain a sense of the overall themes of the interviews, the analysis started with multiple readings of the transcripts along with listening to the audio-recordings. Initial coding comprised highlighting and preliminarily naming important statements. With ongoing discussion and reflection by the research team, early codes were collapsed and categorised creating potential themes which were then applied and reviewed across all transcripts. Further research team reflective discussions then refined these themes names and their narratives into an overarching interpretive metaphor. Metaphors are powerful approaches used in Pacific methodologies to conceptualise research findings into the lived Pacific experience, interpreting the natural environment, key cultural practices, ancient ritual and ceremony of the Pacific world to enable narrative clarity [33,34]. The use of Reflexive Thematic Analysis allowed for the recognition of place and context of our participants, whilst the multiple reflective discussions generating the themes and the metaphor ensured the robustness of the analytical process consistent with Reflexive Thematic Analysis [11,14,22].

### Second stage community and participant consultation

True to the Talanoa research methodology guiding this study, which strongly values reciprocity, respect, humility and love [17] and focuses on building relationships between the researchers and the participants [18], we presented our findings back to participants and their families and communities. Two community gatherings were held to reflect on and discuss our findings; providing a member reflection opportunity for these communities to present further insights [14]. Privileging reciprocity we also explored what participants and families wished us to do with these findings that would benefit them. Both events observed Pacific cultural protocols such as an opening prayer by a Pacific church minister or senior community leader, introductions, activities to nurture the vā [Pacific concept referring to the relational space between people, things, and the environment, signifying connection, respect, and the quality of relationships] and build and strengthen relationships, community acknowledgements, lunch and a closing prayer.

As most participants came from the Auckland region, the first gathering was for participants only and their families. It was held at a church hall which was the venue for a monthly Auckland Pacific Parkinson’s group, and thus a place familiar to many. After the Pacific research team (ML, CS) presented the findings briefly, an open discussion was held, and summary field notes were taken by the team of the key discussion points. For people located further afield, a similar presentation and discussion was held over zoom conferencing.

The second community gathering was held in a larger community venue in South Auckland and open to the Pacific community to attend and was called “Building Strength Through Connection”. Participants living in the South Island of Aotearoa were flown to Auckland to join this second gathering to ensure all were included. Enacting a process of authentic co-design together with member reflections [35], after a brief presentation of the findings, attendees were asked to discuss in smaller groups the following question: *What would have helped when first diagnosed*? The research team facilitated these discussions. Key points, thoughts and ideas were recorded on post-it notes and/or larger paper by either the attendees or the research team. These documentations were collated and summarised to guide the smaller focused participant online talanoa. Participants from our study were provided the opportunity to volunteer for these smaller talanoa. Two online talanoa were held and synthesised and finalised what the team could optimally do to reciprocate the knowledge gained.

## Findings

Of 14 people who responded to our widespread recruitment drive, eight people consented to participate and were interviewed (6 males, 2 females). As seen in Table 1, participants’ ages ranged from 34–73 (mean 55) years with 3 under the age of 50 years. They had experienced symptoms for between 8–29 (mean 16.5) years and had been diagnosed for between 1–28 (mean 17) years. Parkinson medication use ranged from one to six drugs and some participants also lived with other long term health conditions, such as hepatitis, arthritis, hypertension, and diabetes mellitus. We had no access to genetic test results for any participants.

**Table 1:** Participants’ age, duration since first noticed signs and symptoms (S&Ss) and duration since diagnosis.

Participant	Age (years)	Duration since S&Ss start (years)	Duration since Diagnosis (years)
P15	58	29	28
P17	34	14	10
P18	48	13	13
P19	73	10	6
P20	68	28	25
P21	47	10	1
P22	60	8	8
P23	51	24	21

Participants had a range of Pacific ethnicities including Samoan, Tongan, Tokelauan and Fijian; with many being of mixed Pacific ethnicity. All lived with family and had someone to support them, primarily their spouse.

Physical tests were undertaken with six participants, and their Part III scores ranged from 1–46. Falls were a major concern for four participants who were rated as having a high probability of falling in next 6 months, with one participant a moderate and one a low probability. The TUG scores ranged from 6.98–19.53 (mean 10.54) seconds, with only two participants scores greater than the cut-off time for falls risk (>11.5s) established in a sample of people living with typical Parkinson’s (n = 2097) [32].

Collectively, these data demonstrate that our participant sample was diverse with regards to age, years since diagnosis, Pacific ethnicities, physical ability and risk of falls. Our eight participants provided sufficient richness and depth of information to generate meaningful and relational themes that portrayed their journey with Parkinson’s.

### Themes

The Reflective Thematical Analysis generated an overarching theme portrayed by a metaphor of a journey that participants had and were navigating from the time of their Parkinson’s diagnosis. It was evident when participants told their story of their experiences and the impact of their Parkinson’s diagnosis, that it was akin to the sudden start of a new and scary journey, that their life path had unexpectedly changed direction. It was like travelling on an unpredictable wave, and thus it was not just of any journey, but a journey on the high seas in a va’a/vaka (a traditional Polynesian outrigger canoe). This metaphor comprised four themes (1) An unexpected journey, (2) Who’s on the va’a/vaka with me? (3) Navigating your va’a/vaka – looking up to the stars, and (4) Steering your va’a/vaka – finding your way. Below we describe each theme, illustrating this description with participant quotes. We ascribe the quotes to participants, whose anonymity is maintained by the provision of pseudonyms.

#### An unexpected journey.

Participants spoke of the surprise they received by their diagnosis, something they were not expecting and the overwhelming feeling this presented them. How it was a “big” diagnosis, one they realised would impact immensely on both them and their ‘āiga/kāinga/kāiga/vuvale and one they were still coming to terms or not with: “I don’t want to come to terms with this …. I didn’t want this new life.” (P18) The toll on mental health was huge and for some, even suicidally traumatic. P21 spoke of how the diagnosis was simultaneously both a relief and a concern “Like we’re… finally got told it was Parkinson’s. …Ah, relief, ‘cause I finally find… this is what it is. ….We’re just, ah… moving forward … yeah, we don’t really… yeah, it’s such an unanswerable disease. …..You just don’t know how, like… what we need… we don’t know what life is gonna be like.” (P21) P23 lamented that “I think that’s another problem with Pacific people I know, they hardly show up with disease with the Parkinson’s.”

Participants spoke of emotions such as depressive thoughts, anger, embarrassment, devastation, frustration, and anxiety: “I went through a lot of, um... I went through a lot of, sort of like down mood swings. Frustration... depression, anxiety.” (P21) And “I found it really difficult to be around people” (P22). Adding to the stress, participants spoke of their lack of sleep or changes in sleep patterns. P23 spoke of his mood swings, of his anger: “anger, the mood changes every time yea I angry so easily sometimes I growl to my kids as well as my wife”. (P23)

Other participants, in trying to come to terms with their new diagnosis: “I think the only thing that he [neurologist] came out with, is… you, it’s a disease that… you gotta live with it”. (P20) With many taking solace in their spirituality and their deep genealogical connections to tupuna (ancestors) and to the past: “the stories of the past.” (P20) Such ancestral stories often related to brave seafaring journeys. Others had many unanswered questions, reflecting on what this diagnosis meant for them now and into the future “when/how fast are these symptoms going to come”(P22) and “Questions in my head, what’s going to happen now?”(P18) Participants talked of their fear of losing their life or of losing their friends and family.

For participants who were diagnosed with early onset Parkinsons, it was a particularly lonely journey, wanting someone to talk to who was in the same situation, on the same unexpected journey. “Nobody understands it ….. everyone they see me … they see me as abnormal ….. the journey’s been hard to… it’s just a matter of trying to… get people to understand what, what Parkinson’s is, ‘causes it’s still pretty new.” (P18)

#### Who’s on the va’a/vaka with me?.

This journey often felt a solitary and challenging one, which even the person’s family could not fully comprehend: “the journey’s been hard to…it’s just a matter of trying to… get people to understand what…Parkinson’s is”. (P18) At first, the journey was guided by the medical team and was a confusing alienating voyage of different specialists doing different tests and different people offering different support all at different times and in different spaces. As P20 discusses with the interviewer (I):

P20: I can see myself, it’s not a thing… I get… slow… I say… ah, I don’t… I, ah… this is the first time I went to work… ah… I sit under the trees for a rest. And I come back. I can feel myself … when I go for a bath… I can tell, it’s not the same. When I eat, it’s not the same… Then, we went… we went to a, a doctor, who… and, um… he’s the one… and then, when I see him… and he said… took upon what I said, and he said, ah, I’ll refer you to see a specialist. But, when we went to see the specialist… and then he prescribed me the… um, some medicine, eh? And he gave me, ah, Sinemet… Once I, ah, the first time I took the Sinemet… all the things which hold my, holding me back… The stiffness is gone …… And then… from that time, I was… he said, ah you, you’re… you’re sick with Parkinson’s. You’re diagnosed. From the first time I take the medication, eh? I, because… even though I had the, the long-time sick, they, they didn’t prove it, eh.

I: Did you know what Parkinson’s was?

P20: No.

I: Did they explain it to you?

P20: No. …… Only bit they said it’s Parkinson’s … he didn’t explain what it is.

In the early days of diagnosis at least, a supportive team started to develop, although it was difficult to access a specialist, often accentuated by geographical access issues. Participants then began to realise that the best approach was one of specialist care, a team approach of healthcare professionals who knew and understood the individual and what their symptoms were.

Like... most of us with Parkinson’s don’t realise that we need to have a bit of a team.... for the different parts …. ‘Cause not one person, you know, can tell us about all the different bits....and so I’m slowly building myself a team. (P22)

However, once the diagnosis was confirmed and the medications were sorted, the medical team support dwindled and for some, lack of continuity in the health system, causing one participant to lament “but this disease [Parkinson’s] doesn’t end so why do the supports?” (P21). Advocacy from family was important. Once the family understood the condition, they were able to go with their family member to medical visits and ask more questions. However, P21’s wife, who is not of Pacific identity, commented on how it was easier for her to ask questions than her husband:

P21: She’s my advocate.

Wife: Yeah, like sometimes… I don’t know, it’s just the way you are, you just kind of… he, he’s… like, it’s a doctor, and so he’ll…

P21: Mmm, yes sir. [laughs softly]

Wife: Yeah, yeah, but… and then I’m like the one that’s like the Palagi [a white person],

going like, no… he can’t have, and you listen and, and trying to get… been pushing to

get him, like support and stuff.

Having family and friends around became important, P17 commenting that their Parkinson’s “stops me from doing things that I like to do” but that their friends were very supportive and their son accompanies them on trips “to help me with my medication” (P17).

#### Navigating your va’a/vaka – looking up to the stars.

As the journey progressed and less formal healthcare support was offered, participants spoke of how they turned to their communities for support and the safety of places where people knew them. “Cause we went to a mainstream Parkinson’s…We couldn’t see any Pacific Island.” (P20) However, often their families and communities did not understand their condition, having no knowledge of Parkinsons, as P15 comments “I think that’s another problem with Pacific people I know, they hardly show up with disease with the Parkinson.”

Whilst participants and their communities deep connection to the land, tupuna and traditional stories provided comfort and support, it could also cause more stress as some in the community could be disapproving “People judge” and suggests the role of curses and thus be stigmatizing: “my sickness, it’s not… for the outside…that’s like [traditional] medicine… it is for… for the hospital”. (P20)

This conflicting sense between connection and disconnection participants felt in community or social spaces caused a sense of isolation. This was exacerbated for those with early onset Parkinson’s as they were connected with current Parkinson’s support networks where most presented with typical Parkinson’s and thus were older and “looks like it’s only for Palangi [a white person]” (P20). They spoke of how often even the healthcare professionals did not understand early onset Parkinson’s and how it presents.

Sharing and passing on knowledge and information with their families and communities was important “So they don’t have to go through what [this person] went through”. (P21) The importance of knowledge sharing was accentuated by a story told by P17. They first noticed symptoms in the early twenties and although it progressively worsened they did not seek a medical diagnosis. They did “Trying everything, like um, Samoan fofō … um, herbs, drinking herbs” but nothing worked and they finally went to the doctors six years later and were diagnosed with Parkinson’s. Now these two participants were confused and worried about their sons: “Um, just hoping that, I don’t know whether he will get it, or not get it… Just wish that… life for him would be… easier and… hope, just hope that he wouldn’t get it.” (P20) and “Um, just hoping that, I don’t know whether he will get it, or not get it… Just wish that… life for him would be… easier and… hope, just hope that he wouldn’t get it.” (P17)

And there was always hope. Being connected to someone who knew more about the diagnosis and other people in a similar boat may provide better support and options “the fact there’s maybe a pathway, or there might be treatments … might be something targeted to [him]”. (P21) But important was having someone to talk to who you could connect with: “I’d like to have someone to talk to, but not just anybody, you know” (P18) …. “some people you connect with” (researcher)…. “yeah, yeah” (P18).

Participants also drew on the strength of their strong spiritual beliefs “That is why I praise the Lord for the strength and the healing he gives me as well”. (P19) “My faith is a lot stronger now …. For me, when everything is right, you know, in here and up there, everything else follows”. (P22)

#### Steering your va’a/vaka – finding your way.

For many who had navigated the journey for some time, they were finding their own passage, learning to take control over their pathways “I say to the Parkinson’s, ….. I can handle you”. (P19) Having positive mindset was what got them going “This is me and I have a bit of Parkinson’s, this is not me Parkinson’s that has a little bit of me”. (P22) P23 had stronger advice “ahh as I’ve said I’m don’t care you have said to me that you are Parkinson. I don’t care but that’s my advice to those people who are maybe affected by it, don’t be scared you know Parkinson is not a bad disease I think you can handle it you can do ahh whatever you want to do but as long as you got the Parkinson you control it by taking your medication yea.” (P23)

Connecting with other people like you would be, as P21 said “10 million dollars ….. I mean, part of… like just knowing that there’s more people out there helps, I think. … She [Parkinson’s nurse] said there’s one other person with early-onset Parkinson’s … yeah… but … he’s not Pasifika, and it’s not the same…”. (P21) Having people around you that understand Parkinson’s and can support you is crucially important: “I am very thankful for the people around me and close to me they understand my situation. They support my family, and they are aware of myself. My church people are all aware of my condition, that is why of how grateful I am. You know around the community, those people who knows me they are, so they support”. (P23)

Approaches people found helpful included physical activity “You have to do things, yourself, keep moving …. walking when I walk about from here to there I feel my knees tired I sit down when I go shopping, I feel I force to walk sometimes because I want to fight”. (P15) “Once I was diagnosed, I never stopped playing sports.” (P19) Joining a group was motivating “if there’s no group stuff organised, to get me out that door…I will just potter.” (P22) However, the group needs to be one they could relate to, as P23 recounted: “I went there uhmm you know what made me mad, there was just old ladies and old men. I thought that does not match with me”. (P23) P15 found: “I always like exercising … So your muscles keep moving” and finding boxing helpful. P15 added “Once I was diagnosed, I never stopped playing sports.” (P15)

Being mindful of the external world and not too introspective “takes your focus off inwards and focuses your mind outwards” (P22) … “I can contribute something to help other, other people... then it helps me a little bit more to stop thinking about myself and get out.” (P22) This speaks to Pacific values of service to others and the community. Despite the challenges participants faced, they still felt compelled to help and advocate for others.

When asked what they thought was important for other people to know P18 said:

is there any way we can get the, um, of… of what Parkinson’s is out, out to people? … Just so that they, they don’t have to look at us funny, every time they see us. … More understanding, yeah, more understanding, yeah. (P18)

This is important information to share as it enabled peoples with Parkinson’s to access the community. P19 comments: “You know people who got the Parkinson they say when those people come and talk to you and if you are not comfortable when people see them moving around … [and then they stare at you] … yea don’t like it” (P19).

P23 focused on the positives of what could be done “You should have information. Just easy to keep up with the tablets. Keep up with the tablets. And also … they told me not too stress out too much about it. And that my family will be there too”. (P23). He added “I didn’t want to burden anyone else. You know, it was my feelings, yeah, I took it upon myself to care for myself and heal myself and it’s been really helpful so far. …... You know, when I get exercise, I take it. And its no good being lazy. You know your body it gets too weak and you can’t stand up properly and that”. (P23)

#### Connecting in the taulaga/taulanga [harbour].

Our community talanoa, whilst offering an opportunity to gift back our findings to the community for discussion and verification, also presented an opportunity for participants and their ‘āiga/kāinga/kāiga/vuvale to congregate in-person, to meet an network with other Pacific people who were living with Parkinson’s. The first community talanoa verified and strongly supported our findings. The key points of the second larger community event are summarised in Table 2.

**Table 2:** Second Community Talanoa generated information.

*What would have helped when first diagnosed?*	
Reasons	Suggestions
**More information and education at the time of diagnosis**	
• Not only for people with Parkinsons, but for their caregivers, their families and the wider Pacific community • Reduce mental stress, as the uncertainty and lack of understanding causes great deal of mental stress. • Reduce fear for their children – need to use this information to educate future generations	• Videos • Podcasts • Booklets • Information about services available for people with Parkinsons and their families
**Networks of people with Parkinsons and their families**	
• Knowing people of their own age • Greater networks of those who can support people living with Parkinsons • Increased awareness amongst the public • As this disease doesn’t stop – there is a need for on-going support, e.g., physiotherapy and mental health support	• An app: ◦ Can include exercises with prompts like an alarm, reminders ◦ Members only communication channel ◦ Set appointments ◦ Get in touch with others around Aotearoa ◦ Events that are upcoming ◦ Supporters page (for support for the carers) ◦ Fundraising ideas ◦ A facts page containing Parkinson’s information that someone wouldn’t know in small bits so more easily remembered ◦ Fun activities to keep the mind thinking, e.g., Sudoku, work crosswords, painting with number • Pacific Parkinson’s groups in regions other than South Auckland
**Cultural considerations**	
• Health professional training related to Parkinsons in Pacific communities and accompanying cultural competence training • Someone who speaks in Pacific languages, e.g., Tongan/Samoan, to explain Parkinsons to both the person living with Parkinsons and their family • Resources to be translated into Pacific languages • General information be more readily accessible in the media, GP offices, hospitals etc. to help people understand Parkinsons, e.g., like what is currently available for diabetes. An awareness month or day.	• Rap song/ punchy music with positive messaging in Pacific languages • Podcasts • A Health Passport • Checklist (to do list) for when attending Doctor’s appointments

Ongoing Talanoa led to finalising how participants wanted us to gift back their findings. They requested short videos with Pacific Peoples with Parkinson’s telling those newly diagnosed with Parkinson’s things they wish they had been told at the time, culturally relevant information for health professionals and students, and explaining how Parkinson’s has impacted their lives for the families and their communities.

## Discussion

This study sought to explore and gain understanding of the experiences and impact of Parkinson’s on Paciﬁc Peoples living with Parkinson’s and their ‘āiga/kāinga/kāiga/vuvale in Aotearoa. Foremost participants spoke of the sudden and frightening impact their Parkinson’s diagnosis had not only for them personally but for their families and their communities. It was an unexpected diagnosis and a health condition they knew very little about, but one which substantially altered their life course.

The unexpectedness and unfamiliarity of this new life direction (“the unexpected journey”) was perhaps unsurprising, given that the prevalence of Parkinson’s in Aotearoa appears relatively low (160 per 100,000) [4], and thus a health condition that the Pacific population, due to lack of exposure, may know little about. Further, the operative words “appears to be” is used because little is known about Parkinson’s and the Pacific population in Aotearoa. When we recruited for this study, we found that national databases, which we hoped to recruit through, did not at the time collect ethnicity data. Further to this, to date, little has been published on Parkinson’s and Pacific population in Aotearoa. A 2020 scoping review [36] on the variations in prevalence and therapy in Australasia for Parkinson’s across different ethnicities made no comment on Pacific Peoples other than noting the prevalence and incidence data collected via Pitcher et al [4]; their discussion focused on the Māori and Asian populations in Aotearoa and Australia. Pitcher et al [4] calculated prevalence and incidence from the New Zealand Pharmaceutical Collection of prescribing patterns of antiparkinsonian medications in Aotearoa. The lack of research related to Pacific Peoples and Parkinson’s is not only confined to Aotearoa. Siddiqi et al [36] identified in a review of Parkinson’s literature spanning 2000–2024, that only 4.8% (n = 1141) articles included ethno-racial factors as an integral part of the analysis, showing the systematic and global neglect of research related to race and ethnicity in Parkinson’s.

Reasons explaining the currently known prevalence for Pacific Peoples in Aotearoa are speculative, with Pitcher et al [4] suggesting factors such as data quality, inequalities in access to health care, and differing exposure to determinants of Parkinson’s disease. Given our study’s findings it is worth considering the impact access to health care may have. In our community talanoa, participants spoke of the importance of culturally appropriate and safe health care and of health professionals upskilling their competency in how to appropriately deliver care for Pacific Peoples, this aligns with qualitative work completed by Higgs et al. [37]. Neville et al. [38] reported the barriers to older Pacific peoples’ participation in the health-care system in Aotearoa, and detailed four key barriers, namely, getting to and from health care services, cost of services, relationships with health care providers, and not understanding the health-care system. We theorise that the combination of the lack of knowledge about Pacific people with Parkinsons and current barriers to healthcare services for Pacific Peoples may prevent those presenting with the signs and symptoms of Parkinsons seeking health care, and thus the current prevalence may be largely underestimated. There may well be a higher prevalence of Parkinson’s in Aotearoa’s Pacific population than currently known, as research is recently emerging of the PINK1 variant in Polynesians. Case studies are being published of presentations in Tongan and Samoan adults in Aotearoa [7] and in Australia [39]. As this research is new and ongoing it presents an avenue of further attention.

The themes presented in our study of “the unexpected journey” and “who’s on the va’a/vaka with me” are not dissimilar to responses of people newly diagnosed with Parkinson’s in other population groups. An Italian qualitative study identified a similar theme, naming the diagnosis of Parkinson’s as a “reconfiguration of social identity” [40] which spoke to the “turbulent event” and the entrenched tensions of transition, stigmatisation and isolation, and the support required for these “vulnerable identities.” Indeed, the feeling of stigmatisation, shame and embarrassment is often reported by people living with Parkinson’s and identified to arise out of societal attitudes and alternative explanations for Parkinson’s such as ageing, clumsiness, or supernatural causes [41].

Isolation featured prominently in our participants’ deliberations and actions of “who’s on the va’a/vaka with me” and “navigating your va’a/vaka – looking up to the stars”. Whilst isolation is a recognised social outcome of Parkinson’s as the developing symptoms and increasing stigma progressively limit social engagement and intensify social withdrawal [42,43], our participants were proactive, taking deliberate action to build support teams of medical specialist care and of sharing and passing on knowledge and information with their families and communities. The latter was highlighted as important. Given the highly collectivist and family-orientated nature of Pacific culture [44], isolation can be a particularly vulnerable position for many Pacific Peoples. Pacific Peoples’ strong commitment to service is part of what it means to be Pacific. A thread throughout our study was participants’ desire to help others going through the same journey, to contribute to others, and this would have exacerbated their feelings of isolation. Growing the knowledge and understanding of Parkinson’s in their communities is thus crucial to build on the strength within the Pacific culture of community. Strong community networks will then enable strong support and advocacy. Moya-Galé et al. [45] qualitative study in the United States identified the importance of social connectedness for individuals with Parkinson’s through fostering new social connections and community building. As this is a value and way of being deeply rooted in Pacific communities, it is a strength that can thus be easily employed to support those with Parkinson’s within the community.

In “steering your va’a/vaka – finding your way” our participants spoke of how a positive mindset and of hope enabled them to take control of their journey with Parkinson’s. This optimism and hope were facilitated for some by their Christian faith. Spiritual health plays a significant role to support holistic wellbeing for Pacific Peoples [46,47], and again a way of being that may be beneficial as people grapple with the unexpected and scary journey of living with Parkinson’s.

In describing their journeys with Parkinson’s our participants elucidated a torturous journey that started as unexpected and overwhelming but eventuated in them taking the helm and voicing hope and optimism instead of fear and isolation. This analogy reflects that which Gardenhire et al [48] describes in the search of people with Parkinson’s for optimism. Optimism is a protective factor for good mental health, promoting resiliency and reducing distress in health crises [49]. In their grounded theory study, Gardenhire et al [48] reported that people living with Parkinson’s grow their optimism in the face of challenges by reframing optimism as a choice rather than a feeling. Their participants distinguished “feeling optimism” (emotions) from “doing optimism” (behaviours). In “steering your va’a/vaka – finding your way” our participants were taking control of their journey and being mindful of the external world, taking actions that Gardenhire et al [48] labels as “doing optimism”. This, these authors say, includes a sense of mastery in other areas of their life such as lifestyle changes, (for example some of our participants spoke of engaging in physical activity) and advocating for other people with Parkinson’s (one of our participants advised against being too introspective, and our community talanoa requested more resources to drive community knowledge and understanding). Gardenhire et al [48] do however warn that to get to “doing optimism”, people must traverse the process of accepting their diagnosis and this emotional process needs time for adjustment and to find a place of “feeling optimism”.

Our participants said they found engagement in physical exercise helpful. There is substantial evidence of the benefits of physical exercise on the severity of motor signs and quality of life of Parkinson’s [50], prompting the call for exercise to be considered as medicine in Parkinson’s and prescribed as an adjunct treatment as early as possible [51]. However, for Pacific Peoples, physical activity is deeply entwined with culture, community, spirituality, and identity, commingling everyday living, social connection, and cultural expression. Cultural values, gender roles, and the importance of social networks thus shape how people engage in physical activity [37,52]. An exploration of physical activity for Sāmoan stroke survivors challenged the value of the Western approach of medicalising physical activity, reducing it to a set of prescribed exercises of physical tasks, and proposed a holistic and relational approach integrating the spiritual, emotional, and social aspects of wellbeing [53]. This proposition could be extended to Pacific Peoples living with Parkinson’s.

Traversing the acceptance of a diagnosis of Parkinson’s and processing the associated emotions, is something one of our participants labelled as the “big diagnosis” in the theme of “an unexpected journey”. This journey caused a huge mental toll, which for some it was suicidally traumatic. Demoralisation can be common amongst people following their “big” diagnosis, compounded by frustration of the persistent and debilitating symptoms of Parkinson’s, loss of hope and feelings of stigma and isolation [54]. Emotions all our participants identified. An inter-disciplinary, holistic and proactive person-centered approach is advocated to manage demoralization, and this includes peer support [54]. Validating the person’s experience of demoralization and compassion are important during the person’s journey, and whilst our analysis presented this journey with a linear analogy, it must be acknowledged that the journey is turbulent, even for a sturdy va’a/vaka. Family, community and health care professionals need to be there to support at critical touch points along the way to drive the sense of hopelessness to one of hope [54].

What was disappointing for our participants was the early withdrawal of medical support – “this disease doesn’t end so why do the supports?” The progressive and permanent nature of Parkinson’s appeared not considered by the healthcare services, leaving feelings of abandonment, and thus perpetuating demoralisation. This is analogous to the question posed by Longmuir et al. [55] regarding the preservation of hope and promotion of meaningful lives for those with Duchenne muscular dystrophy (DMD). This latter study suggested that “the moral value of each life lived with DMD should be about enabling people’s lives to have purpose and meaning, to be included in society, and to be able to participate in, and contribute to, their communities” [55 p.20]. The same could be said for those living with Parkinson’s – hope enables meaningful lives; healthcare services should morally value lives over social exclusion based on neoliberal policies and discourses that values fiscal savings over meaningful and valued lives [55].

### Clinical implications for healthcare services

This study highlights important clinical messages for healthcare services and organisations supporting people with Parkinson’s. Firstly, collecting ethnicity data is important to both address health equity and deliver culturally appropriate healthcare [56]. This leads to the second key message; the crucial positive impact health professional cultural competence has on health outcomes [56]. Cultural influences have a direct effect on health outcomes; thus, the healthcare work force need to understand and value the realities of Pacific individuals, families and communities so that accessible, appropriate, safe, and effective care can be provided for Pacific Peoples living with Parkinson’s [57]. Indeed, cultural competence is a mandatory requirement of registered health professionals in Aotearoa, and Aotearoa appropriate cultural models of health care are now taught in tertiary education institutes [58–60]. The third message is that people need ongoing health care support to live well with this lifelong condition that impacts all aspects of a person and their families’ lives. Whilst “the diagnosis” is important, it is only the beginning of the journey; possibly more important is the continuity of support from the health system and its services and reduction in the feeling of abandonment. Continuation of health care services is especially important when working with Pacific Peoples. This important consideration is linked with our fourth key message that health professionals working with Pacific Peoples with Parkinson’s should be cognisant of the possibility of the PINK1 variant. Although we did not have access to generic profiling for those who may have been tested, consideration of our participant demographic data showed that many had early onset Parkinson’s. Six participants were under the age of 50, and one participant was 52 years of age, when their Parkinson’s signs and symptoms first appeared. Some participants commented that their healthcare professionals and the community support groups did not understand about their early onset and presentation. Whilst not much is currently known [61], the PINK1 variant appears characterised by an early-onset, a slow progression and lower limb dystonia, and thus its management may differ from those diagnosed with late-onset idiopathic disease [62]. This is crucial to understand given the long-term condition burden of those presenting with early onset Parkinson’s.

### Reciprocity

Mindful that a key research principle of this study was one of reciprocity, our community talanoa identified ways our communities wished for the study findings to be used. As Parkinson’s was perceived to be a “new” condition for Pacific communities, the main request was for us to develop short informative videos. We are currently videoing interviews with consenting participants about what they think other Pacific People newly diagnosed with Parkinson’s and the public ought to know and their key messages for health professionals and health professional students. It was considered important that these messages come from Pacific People with Parkinson’s themselves as people trust those who share similar cultural values and beliefs [63]. Our participants asked us to share these videos publicly on a Aotearoa health information website, Healthify (previously known as Health Navigator) trusted by both the public and health professionals [64].

### Strength and limitations

To the best of our knowledge this is the first qualitative study to explore the experiences and impact of Parkinson’s on Paciﬁc Peoples living with Parkinson’s and their ‘āiga/kāinga/kāiga/vuvale in Aotearoa. A strength of our study was that it was underpinned by Pacific values and all key research team members identifying as of Pacific heritage. Despite a widespread recruitment strategy, only 14 people responded. The eight participants were those who responded to in-person approaches by others on our behalf where trusted relationships already existed. Although we addressed many factors to enhance recruitment, such as Pacific researchers, use of Pacific methodologies, targeted recruitment techniques and translated recruitment material, key to participation were these trusted relationships and partnership building [65]. That said, whilst our sample size was small and heterogeneous, we achieved theoretical saturation, and our participant sample was diverse with regards to age, years since diagnosis, Pacific ethnicities, physical ability and risk of falls. Most participants, however, lived in the North Island of Aotearoa, where most of the Pacific population reside.

## Conclusion

We explored the experiences and impact of Parkinson’s on Paciﬁc Peoples living with Parkinson’s and their ‘āiga/kāinga/kāiga/vuvale in Aotearoa. We found the impact of their Parkinson’s diagnosis was momentous, unexpectedly and permanently changing their life path direction. This new life-journey was compared to travelling on an unpredictable wave on the high seas in a va’a/vaka. Initially, participants felt overwhelmed and experienced a range of emotions such as depressive thoughts, anger, embarrassment, devastation, frustration, and anxiety. In the early stages, support was offered by the medical team but then this support dwindled and participants had to turn to their family and community for support. But as Parkinson’s is not a well-known health condition in this population, participants found that they had to be proactive and find their own ways to navigate their lives with Parkinson’s. They drew strength from their spirituality and their deep genealogical connections to tupuna (ancestors) and to the past, from their families and communities, and this adjusted their sentiments to those of hope and optimism.

Given the limitations of Aotearoa’s current health care systems, and the low number of health care specialists, a supportive outrigger for the va’a/vaka is even more crucial than ever, and for Pacific communities this most likely comes from the family and the community. For this support to develop in what is a “new” health condition for Pacific Peoples, growing knowledge and understanding of Parkinson’s in ways that are accessible and acceptable to families and the community is essential. Added to this is the need for healthcare services to improve how they offer accessible care and support Pacific communities in culturally appropriate and safe ways that considers the permanent and progressive nature of the condition.

## Supporting information

S1 Checklist Questionnaire on inclusivity in global research. (DOCX)

## References

[1] Poewe W, Seppi K, Tanner CM, Halliday GM, Brundin P, Volkmann J, et al. Parkinson disease. Nat Rev Dis Primers. 2017;3:17013. doi: 10.1038/nrdp.2017.13 28332488

[2] GBD 2015 Neurological Disorders Collaborator Group. Global, regional, and national burden of neurological disorders during 1990-2015: a systematic analysis for the Global Burden of Disease Study 2015. Lancet Neurol. 2017;16(11):877–97. doi: 10.1016/S1474-4422(17)30299-5 28931491 PMC5641502

[3] Tanner CM, Ostrem JL. Parkinson’s Disease. N Engl J Med. 2024;391(5):442–52. doi: 10.1056/NEJMra2401857 39083773

[4] Pitcher TL, Myall DJ, Pearson JF, Lacey CJ, Dalrymple-Alford JC, Anderson TJ, et al. Parkinson’s disease across ethnicities: A nationwide study in New Zealand. Mov Disord. 2018;33(9):1440–8. doi: 10.1002/mds.27389 30035822

[5] Health and Disability System Review – Final Report – Pūrongo Whakamutunga. Wellington: HDSR. 2020. https://www.health.govt./system/files/2022-09/health-disability-system-review-final-report.pdf

[6] Ministry of Health and Ministry of Pacific Island Affairs, Tupu Ola Moui: Pacific Health Chart Book. Wellington: Ministry of Health; 2004. https://www.health.govt./system/files/2022-09/pacific-persepectives-health-system-review-final-pdf-version.pdf

[7] Patel SG, Buchanan CM, Mulroy E, Simpson M, Reid HA, Drake KM, et al. Potential PINK1 founder effect in Polynesia causing early-onset Parkinson’s disease. Mov Disord. 2021;36(9):2199–200. doi: 10.1002/mds.28665 34159639

[8] Donnelly J, Buchanan C, Patel S, Vailahi G, Viali S, Puli’uvea C, et al. PINK1 Parkinson’s Disease in New Zealand: An overview. Neurol. 2025;104(7 Suppl 1). doi: 10.1136/bmjno-2025-ANZAN.130

[9] Parkinson’s UK. https://www.parkinsons.org.uk/information/symptoms

[10] Parkinson’s NZ. https://www.parkinsons.org.nz/understanding-parkinsons/what-is-parkinsons

[11] Braun V, Clarke V. Using thematic analysis in psychology. Qual Res Psychol. 2006;3(2):77–101.

[12] Braun V, Clarke V. One size fits all? What counts as quality practice in (reflexive) thematic analysis? Qual Res Psychol. 2021;18(3):328–52. doi: 10.1080/14780887.2020.1769238

[13] Braun V, Clarke V. Conceptual and design thinking for thematic analysis. Qual Psychol. 2022;9(1):3–26. doi: 10.1037/qup0000196

[14] Braun V, Clarke V. Toward good practice in thematic analysis: avoiding common problems and be(com)ing a knowing researcher. Int J Transgend Health. 2022;24(1):1–6. doi: 10.1080/26895269.2022.2129597 36713144 PMC9879167

[15] Sopoaga F, Nada-Raja S, Leckie T, Samaranayaka A, Covello S. Mental Health and well-being of first year pacific students at a New Zealand university. Open J Soc Sci. 2023;11(07):365–81. doi: 10.4236/jss.2023.117026

[16] Vaioleti TM. Talanoa research methodology: a developing position on Pacific research. Waikato J Educ. 2016. doi: 10.15663/wje.v12i1.296

[17] Enari D. Methodology marriage: merging Western and Pacific research design. Proc J Interdiscip Res. 2021. doi: 10.26021/10641

[18] Ravulo J. Creating a mental health talanoa to promote a collaborative approach to wellbeing across Pacific peoples. Australas Psychiatry. 2025;33(2):207–9. doi: 10.1177/10398562241281576 39311430 PMC11982575

[19] Naepi S. Pacific research methodologies. In: Oxford research encyclopedia of education. 2019. doi: 10.1093/acrefore/9780190264093.013.566

[20] Rangiwai B, Enari D, Masae C, Paea D, Tahilanu-Mapili L, Vailahi V. Lost in translation: reflexive thematic analysis in research with Pacific peoples. tekaharoa. 2021;17(1). doi: 10.24135/tekaharoa.v17i1.354

[21] Kidder LH, Fine M. Qualitative and quantitative methods: When stories converge. In: Mark MM, Shotland L, editors. New directions for program evaluation. Jossey-Bass; 1987. pp. 57–75. doi: 10.1002/ev.1459

[22] Braun V, Clarke V. Supporting best practice in reflexive thematic analysis reporting in Palliative Medicine: A review of published research and introduction to the Reflexive Thematic Analysis Reporting Guidelines (RTARG). Palliat Med. 2024;38(6):608–16. doi: 10.1177/02692163241234800 38469804 PMC11157981

[23] Braun V, Clarke V. Successful qualitative research: A practical guide for beginners. Sage; 2013.

[24] Koya-Vaka’uta CF. Rethinking research as relational space in the Pacific: Pedagogy and praxis. In: Vaai UL, Casimira A, editors. Relational Hermeneutics: Decolonising the Mindset and the Pacific Itulagi. Suva: University of the South Pacific, The Pacific Theological College; 2017. pp. 65–84.

[25] Leenen-Young M, Uperesa L. Re-visioning Pacific research method/ologies. J Polyn Soc. 2023;132(1/2):9–39. doi: 10.15286/jps.132.1-2.9-39

[26] O’Brien BC, Harris IB, Beckman TJ, Reed DA, Cook DA. Standards for reporting qualitative research: a synthesis of recommendations. Acad Med. 2014;89(9):1245–51. doi: 10.1097/ACM.0000000000000388 24979285

[27] Hoehn MM, Yahr MD. Parkinsonism: onset, progression and mortality. Neurology. 1967;17(5):427–42. doi: 10.1212/wnl.17.5.427 6067254

[28] Goetz CG, Tilley BC, Shaftman SR, Stebbins GT, Fahn S, Martinez-Martin P, et al. Movement Disorder Society-sponsored revision of the Unified Parkinson’s Disease Rating Scale (MDS-UPDRS): scale presentation and clinimetric testing results. Mov Disord. 2008;23(15):2129–70. doi: 10.1002/mds.22340 19025984

[29] Podsiadlo D, Richardson S. The timed “Up & Go”: a test of basic functional mobility for frail elderly persons. J Am Geriatr Soc. 1991;39(2):142–8. doi: 10.1111/j.1532-5415.1991.tb01616.x 1991946

[30] Campbell C, Rowse J, Marcia CA, Shumway-Cook A. The effect of cognitive demand on Timed Up and Go performance in older adults with and without Parkinson Disease. J Neurol Phys Ther. 2015;27(1):2–7. doi: 10.1097/01253086-200327010-00002

[31] Paul SS, Canning CG, Sherrington C, Lord SR, Close JCT, Fung VSC. Three simple clinical tests to accurately predict falls in people with Parkinson’s disease. Mov Disord. 2013;28(5):655–62. doi: 10.1002/mds.25404 23450694

[32] Nocera J, Stegemöller EL, Malaty IA, Okun MS, Marsiske M, Hass CJ, et al. Using the Timed Up and Go test in a clinical setting to predict falling in Parkinson’s Disease. Arch Phys Med Rehabil. 2012;94:1300–5. doi: 10.1016/j.apmr.2013.02.020 PMC4144326 23473700

[33] Powell EN. Tei te akau roa: an ocean of metaphor in Pacific research methodologies. J Polyn Soc. 2023;132(1/2):41–56. doi: 10.15286/jps.132.1-2.41-56

[34] Goodwin DWT, Boulton A, Stayner C, Mann J. Authentic co-design: an essential prerequisite to health equity in Aotearoa New Zealand. J R Soc N Z. 2025;55(6):2600–14. doi: 10.1080/03036758.2025.2480207 40756847 PMC12315192

[35] Alamri Y, Pitcher T, Anderson TJ. Variations in the patterns of prevalence and therapy in Australasian Parkinson’s disease patients of different ethnicities. BMJ Neurol Open. 2020;2(1):e000033. doi: 10.1136/bmjno-2019-000033 33681780 PMC7871730

[36] Siddiqi S, Ortiz Z, Simard S, Li J, Lawrence K, Redmond M, et al. Race and ethnicity matter! Moving Parkinson’s risk research towards diversity and inclusiveness. NPJ Parkinsons Dis. 2025;11(1):45. doi: 10.1038/s41531-025-00891-7 40050644 PMC11885646

[37] Higgs C, Taungapeau F, Silcock C, Sanerivi O, Fruean E, Lameta I, et al. Holistic health for Pacific seniors from a weekly group gathering run by a Pacific health provider. J Prim Health Care. 2023;15(4):358–65. doi: 10.1071/HC23093 38112710

[38] Neville S, Wrapson W, Savila F, Napier S, Paterson J, Dewes O, et al. Barriers to older Pacific peoples’ participation in the health-care system in Aotearoa New Zealand. J Prim Health Care. 2022;14(2):124–9. doi: 10.1071/HC21146 35771708

[39] Morales-Briceno H, Ong TL, Duma SR, Murray N, Pepper EM, Ha A, et al. Recurrent Biallelic p.L347P PINK1 Variant in Polynesians with Parkinsonism and Isolated Dopa-Responsive Dystonia. Mov Disord Clin Pract. 2022;9(5):696–7. doi: 10.1002/mdc3.13467 35844286 PMC9274338

[40] Landolfi L. Social identity of people with Parkinson’s disease: an interpretation of the disease. Ital Sociol Review. 2025;15(3):625–47. doi: 10.13136/isr.v15i3.1008

[41] Crooks S, Mitchell G, Wynne L, Carter G. Exploring the stigma experienced by people affected by Parkinson’s disease: a systematic review. BMC Public Health. 2025;25(1):25. doi: 10.1186/s12889-024-21236-8 39754132 PMC11697948

[42] Ahn S, Springer K, Gibson JS. Social withdrawal in Parkinson’s disease: a scoping review. Geriatr Nurs. 2022;48:258–68. doi: 10.1016/j.gerinurse.2022.10.010 36332441 PMC9742332

[43] Perepezko K, Hinkle JT, Shepard MD, Fischer N, Broen MPG, Leentjens AFG, et al. Social role functioning in Parkinson’s disease: a mixed-methods systematic review. Int J Geriatr Psychiatry. 2019;34(8):1128–38. doi: 10.1002/gps.5137 31069845 PMC6949188

[44] Almao C, Ioane J. Working with Pasifika communities in Aotearoa New Zealand: Through the lens of non-Pasifika psychologists. JCCP. 2023;33(1):92–106. doi: 10.5281/zenodo.8187853

[45] Moya-Galé G, Ahson S, Gyawali S, Lee C, O’Riordan C, Rossi L. Stronger together: a qualitative exploration of social connectedness in Parkinson’s disease in the digital era. Am J Speech Lang Pathol. 2025;34(1):281–96. doi: 10.1044/2024_AJSLP-24-00246 39546420

[46] Hitti PR. Masters of Public Health in Public Health, Pacific peoples’ perspectives on spiritual health. Massey University, Wellington, New Zealand. 2024 https://mro.massey.ac./server/api/core/bitstreams/4a4bd712-5645-4dde-9137-4a1f00f74142/content

[47] Neville S, Wrapson W, Savila F, Napier S, Dewes O, Clair VW-S, et al. The influence of spirituality and religion on health and well-being for older Pacific People. J Relig Spiritual Aging. 2023;36(3):293–308. doi: 10.1080/15528030.2023.2221872

[48] Gardenhire J, Mullet N, Fife S. Living with Parkinson’s: the process of finding optimism. Qual Health Res. 2019;29(12):1781–93. doi: 10.1177/1049732319851485 31179832

[49] Allison PJ, Guichard C, Gilain L. A prospective investigation of dispositional optimism as a predictor of health-related quality of life in head and neck cancer patients. Qual Life Res. 2000;9(8):951–60. doi: 10.1023/a:1008931906253 11284214

[50] Ernst M, Folkerts A-K, Gollan R, Lieker E, Caro-Valenzuela J, Adams A, et al. Physical exercise for people with Parkinson’s disease: a systematic review and network meta-analysis. Cochrane Database Syst Rev. 2023;1(1):CD013856. doi: 10.1002/14651858.CD013856.pub2 36602886 PMC9815433

[51] Langeskov-Christensen M, Franzén E, Grøndahl Hvid L, Dalgas U. Exercise as medicine in Parkinson’s disease. J Neurol Neurosurg Psychiatry. 2024;95(11):1077–88. doi: 10.1136/jnnp-2023-332974 38418216

[52] Enoka P, Hale L, Higgs C. Facilitators and barriers to physical activity for people of Pacific heritage. NZJP. 2022;50(1):33–41. doi: 10.15619/NZJP/50.1.04

[53] Viali ES. Exploring the meaning and value of physical activity for Sāmoan stroke survivors in New Zealand. MPhty thesis, University of Otago. 2025. Aavilabl from: https://hdl.handle.net/10523/47737

[54] McDaniels B, Bradley ER, Pontone G, Pushparatnam K, Mathur S, Miller R, et al. From hopelessness to hope: addressing demoralization in Parkinson’s disease. Mov Disord Clin Pract. 2026;13(1):71–9. doi: 10.1002/mdc3.70209 40590251 PMC12839486

[55] Longmuir K, Park J, Fitzgerald R, Legge M, Shore C. Hope: valuing lives and persons with degenerative conditions – Duchenne muscular dystrophy. Sites: New Series. 2023;20(1):1–26. doi: 10.11157/sites-id526

[56] Loring B, Reid P, Curtis E, McLeod M, Harris R, Jones R. Ethnicity is an evidence-based marker of need (and targeting services is good medical practice). N Z Med J. 2024;137(1603):9–13. doi: 10.26635/6965.e1603 39326017

[57] Tukuitonga C. Impact of culture on health. Pac Health Dialog. 2018;21(1):5–7. doi: 10.26635/phd.2018.901

[58] Tiatia-Seath J. The importance of Pacific cultural competency in healthcare. Pac Health Dialog. 2018;21(1):8–9. doi: 10.26635/phd.2018.909

[59] Tipene-Leach D, Simmonds S, Carter M, Haggie H, Mills V, Lyndon M. Cultural safety and the medical profession in Aotearoa New Zealand: a training framework and the pursuit of Māori health equity. N Z Med J. 2024;137(1607):87–97. doi: 10.26635/6965.6732 39666888

[60] Curtis E, Loring B, Jones R, Tipene-Leach D, Walker C, Paine S-J, et al. Refining the definitions of cultural safety, cultural competency and Indigenous health: lessons from Aotearoa New Zealand. Int J Equity Health. 2025;24(1):130. doi: 10.1186/s12939-025-02478-3 40346663 PMC12063315

[61] Lange LM, Fang Z-H, Makarious MB, Kuznetsov N, Atterling Brolin K, Ballard S, et al. Parkinson’s disease genetics across diverse ancestries: an observational genetic study of causal and risk variants with translational implications. Lancet Neurol. 2026;25(8):741–54. doi: 10.1016/S1474-4422(26)00198-5 42456684 PMC13446284

[62] Yin EP, Dieriks BV. Rethinking “rare” PINK1 Parkinson’s disease: a meta-analysis of geographical prevalence, phenotypic diversity, and α-synuclein pathology. J Parkinsons Dis. 2025;15(1):41–65. doi: 10.1177/1877718X241304814 39973502 PMC13347432

[63] Moore C, Coates E, Watson A, de Heer R, McLeod A, Prudhomme A. Correction to: “It’s Important to Work with People that Look Like Me”: Black Patients’ Preferences for Patient-Provider Race Concordance. J Racial Ethn Health Disparities. 2023;10(5):2614. Erratum in: J Racial Ethn Health Disparities. 2023 Oct;10(5):2614; doi: 10.1007/s40615-022-01489-y 36536166 PMC11837169

[64] Health Quality and Safety Commission. Links to consumer engagement centres: Health Navigator New Zealand. 2022. https://www.hqsc.govt./consumer-hub/engaging-consumers-and-whanau/links-to-consumer-engagement-centres/

[65] Glover M, Kira A, Johnston V, Walker N, Thomas D, Chang AB, et al. A systematic review of barriers and facilitators to participation in randomized controlled trials by Indigenous people from New Zealand, Australia, Canada and the United States. Glob Health Promot. 2015;22(1):21–31. doi: 10.1177/1757975914528961 24842989

