# Supplementary material for: Experiences of Pacific Peoples and their ‘āiga/kāinga/kāiga/vuvale living with Parkinsons: A qualitative study
Source: PLoS One. 2026 Aug 12;21(8):e0355989. doi: 10.1371/journal.pone.0355989 (PMC13465971; doi:10.1371/journal.pone.0355989)
Supplement: Supplementary file 3 [file pone.0355989.s004.docx]

**PONE-D-26-01951 Authors Response to Reviewers' Comments**

Thank you to the reviewers who have taken time and consideration to review our revised version of our paper. This has enabled us to strengthen our paper further. Below we respond to each comment and in our revised paper we have highlighted the changes made in yellow.

**Reviewer #1: Regarding the response:**
"As is usual in qualitative research, some participants were verbose and some did not say very much (P15 and P17 fall into this latter category)". It is still important to include quotes from all participants in qualitative papers, and especially since the thematic analysis suggests that they would have all said something that would have reflected that specific point, there should not have been an issue selecting quotes from all the participants.

Authors’ Response: Please note that in our first revised manuscript we did have quotes from all participants, but we have now added more quotes from P15 and P17.

**Reviewer #2: Thanks for the revision. The authors answered each question briefly; however, the major concerns have not been meaningfully resolved.**

Authors’ Response: We are sorry if our previsions revisions were a little sparse. We have now revised further by expanding our explanations in the manuscript to each of your points to provide greater clarity (see yellow highlighted text).

Methodological approach: Under ‘Design’: see lines 119-128.

**Reviewer #2:** The study still relies on a very small and clinically heterogeneous sample, without adequately justifying how such limited data can support the current thematic structure.

Authors’ Response: Under ‘Participants and Recruitment’: see lines 157-160 and ‘Strength and Limitations’: see lines 707-717.

**Reviewer #2:** In addition, the clinical composition of the sample remains insufficiently defined,

Authors’ Response: We are not sure what more we can add here – we have clinically defined our sample (see lines 260-285, Table 1) and we have discussed under ‘Strengths and limitations’ (lines 713-717). Given that we had 8 participants, many who came from the same area, we could not be more specific in our reporting (such as a more detailed participant demographic table) without identifying our participants and thereby contravening our ethical approval.

**Reviewer #2:** The description of the analysis is still not fully consistent with reflexive thematic analysis,

Authors’ Response: Under ‘Data analysis’: see lines197-228.

**Reviewer #2:** The boundary between primary interview data and later community “verification” remains unclear,

Authors’ Response: Under ‘Second stage community and participants consultation’: see lines 220-228

**Reviewer #2:** The handling of multilingual data is still underexplained.

Authors’ Response: Under ‘Data collection’ lines186-188 and then Under ‘Data analysis’: see lines 197-203.

**Reviewer #2:** Overall, although the revision improves presentation, it does not materially strengthen the manuscript’s methodological rigor.

Authors’ Response: We hope that we have now strengthened our manuscript’s methodological rigour, underpinned by our non-positivist ‘Big Q’ Qualitative research approach as opposed to a post-positivist “q” approach as referenced. (Kidder LH, Fine M. 1987. Qualitative and quantitative methods: When stories converge. In M. M. Mark & L. Shotland (Eds.), New directions for program evaluation (pp. 57–75). Jossey-Bass. doi: 10.1002/ev.1459; Braun V, Clarke V. Supporting best practice in reflexive thematic analysis reporting in Palliative Medicine: A review of published research and introduction to the Reflexive Thematic Analysis Reporting Guidelines (RTARG). Palliative Med., 2024;38(6):608-616; doi: 10.1177/02692163241234800; Braun V, Clarke V. 2013. Successful qualitative research: A practical guide for beginners. Sage.)
