# Supplementary material for: Experiences of Pacific Peoples and their ‘āiga/kāinga/kāiga/vuvale living with Parkinsons: A qualitative study
Source: PLoS One. 2026 Aug 12;21(8):e0355989. doi: 10.1371/journal.pone.0355989 (PMC13465971; doi:10.1371/journal.pone.0355989)
Supplement: Supplementary file 4 [file pone.0355989.s003.docx]

**Revision of manuscript PONE-D-26-01951:**

Thank you to the reviewers for their time and careful consideration our manuscript and for their comments and recommendations. Below we address each of these in turn. Changes in the manuscript are highlighted in yellow.

**REVIEWER #1:** This manuscript is an important contribution to the PD community to enhance diversity in the representation of PD voices. It is a solid step towards understanding the issues faced by the Pacific People PD patients, and hopefully improve the awareness and support for the patients and their families. I am sure there was a lot of data collected during the interviews which was difficult to distil for this manuscript, however the manuscript would benefit from additional information to help the readers understand the quotes and findings more clearly.

***Response:*** Thank you for your positive comments about. and support for. our study. Below we address each of your comments.

Major points:

1. I could not find a detailed demographic table of the participants, only the generalised summary of their ages and conditions. It would be good to include this, without giving away too much personal identifiable detail.

***Response:*** We have added a demographic table of age, duration since first noticed S&Ss and time since diagnosis. We do not wish to add other identifying information such as gender, PINK1 gene diagnosis or Pacific ethnicity to maintain anonymity in what is a small population that mostly know each other. We consider that the information provided in this table will provide sufficient context to the themes and quotes.

2. There were 8 interviewees but I could only find quotes for 5 (P1, P18, P21, P22, P23 and P24). Could the authors include quotes from the other interviewees as well? It seemed as if the majority of the quotes stated come from P18 and P22, it would be good if a more equal distribution was given to the others.

***Response***: We have included more quotes from more participants as suggested. As is usual in qualitative research, some participants were verbose and some did not say very much (P15 and P17 fall into this latter category). We now have a few quotes from all participants. (P15=2; P17=1; P18=7; P19=4; P20=7; P21=7; P22=8; P23=7)

3. The questions in the interview guide were very insightful, but I was not able to see where the answers for some of the important questions like Did you seek medical advice and what was you experience with seeking medical advice? (except for one quote by P22) as this could have direct impact on how the local medical community could be better educated when discussing the PD diagnosis with the patients and their families.

***Response:*** We have added two more quotes related to this topic, but as it fitted in within our theming.

4. The statement 'Advocacy from family was important' could be expanded further.

***Response***: We have expended on this statement and added an extra quote.

5. If possible, it would be good to add more quotes of positive perspectives, especially for the section "Steering your va’a/vaka – finding your way", this would be also a nice way to highlight the resilience of the participants and begin to tease out some of the meaningful and innovative changes/mindset they have developed as their lived experience is a precious resource for others to learn from.

***Response***: We have added to this section as suggested.

Minor Comments:

1. The word Parkinson's should be referred to as Parkinson's disease or PD in the intro/methods etc as this is its proper term, but can be kept as is if this is the term that was used by the participants' quotes.

***Response***: We appreciate your comment, but contemporary nomenclature, that people living with the conditions have specified to use (including in NZ), is “Parkinson’s”. To quote the Parkinson’s UK website: “What is ‘Parkinson’s disease’? …. Some, including healthcare professionals and people living with the condition, will say Parkinson’s disease, or PD for short. We call it Parkinson’s. We don’t use the word ‘disease’ because some people with Parkinson’s have told us it sounds negative, or like an infectious illness. But unlike the flu or measles, you can't catch Parkinson's from someone. We don't yet know exactly why people get Parkinson's. Researchers think it's a combination of age, genetic, and environmental factors that cause the dopamine-producing nerve cells to die. But they agree Parkinson's is not infectious, so we avoid the term ‘disease’.”

So, we would prefer to use the term “Parkinson’s throughout”, unless Reviewer 1 and the Editor feel very strongly about it. We have added a paragraph at the end of the introduction to explain this.

2. It is good that the authors relayed their findings to the community as an engagement exercise, it is not clear whether there is an local association/society/regional chapters through which more activities can be initiated. They may want to include this as a means to highlight the work they do.

***Response***: Thank you for this comment. On page 6 of paper under “Participants and Recruitment’ we noted that we recruited via the Parkinson’s NZ and Parkinson’s Canterbury, so the local association is well aware of our research.

**REVIEWER # 2:** This manuscript presents a qualitative exploration of the experiences of Pacific Peoples living with Parkinson’s disease in Aotearoa New Zealand using Talanoa methodology and reflexive thematic analysis. The study addresses an important and under-researched population and provides culturally grounded insights into the lived experiences of Parkinson’s disease among Pacific communities.
Overall, the manuscript is technically sound, and the qualitative design is appropriate for the research question. The use of the Talanoa methodology and reflexive thematic analysis is well justified, and the involvement of Pacific researchers strengthens the cultural validity of the research approach. The themes identified are coherent and supported by illustrative participant quotations. The study contributes valuable insights into culturally informed care and the social context of Parkinson’s disease. However, several issues should be addressed to strengthen the manuscript.

***Response***: Thank you for your positive comments, much appreciated. We address your issues below.

1. The study includes eight participants. While this sample size is acceptable for qualitative research, the authors should clarify how they assessed thematic sufficiency or saturation. A short explanation regarding how the research team determined that the dataset was adequate for thematic development would strengthen methodological transparency.

***Response***: Our chosen approach to data analysis was that of reflexive thematic analysis, and this approach, alongside our methodology of talanoa, is not coherent with the post-positivism concepts of thematic sufficiency or saturation. We have added a more coherently appropriate statement to page 11, namely: “Our eight participants provided sufficient richness and depth of information to generate meaningful and relational themes that portrayed their journey with Parkinson’s.”

2. Although the study is primarily qualitative, several clinical assessments were collected (e.g., MDS-UPDRS III, TUG, fall risk indicators). The rationale for including these quantitative measures should be clarified. At present, they are only briefly summarized and do not clearly convey the qualitative findings. The authors may either better explain their contextual purpose or consider relocating them to supplementary information.

***Response***: To explain the contextual inclusion of these quantitative data, we have added the following to page 11: “Collectively, these data demonstrate that our participant sample was diverse with regards to age, years since diagnosis, Pacific ethnicities, physical ability and risk of falls.”

3. The discussion on social isolation could be strengthened by incorporating evidence on functional outcomes in PD. Social isolation and reduced community participation were evident in participants’ narratives. Social functioning is increasingly recognized as an important outcome in PD, as motor symptoms, cognitive changes, and stigma may limit engagement in social roles and daily activities (Ahn et al., 2022; Su et al., 2020; Yu et al., 2022). Refs: Ahn et al. Geriatric Nursing 48 (2022): 258-268. Su et al. Journal of Parkinson’s Disease 10.3 (2020): 1143-1151.;Yu et al. Frontiers in aging neuroscience 14 (2022): 913958.

***Response***: Thank you for drawing our attention to this work – we have included the impact of social isolation into our discussion – see page 26.

4. The discussion appropriately links findings to existing qualitative literature on Parkinson’s disease and stigma. However, the authors may strengthen the manuscript by More clearly articulating the clinical implications for healthcare services. In addition, highlighting how culturally responsive care models could be implemented in practice. Moreover, clarifying how the proposed community resources (videos, educational materials, etc.) will be translated into health service improvements.

***Response***: Yes, a good point. We have included a section in the discussion on clinical implications and implementation of health care models. And we have addressed your translation of health service improvements. Please see pages 29-30.
