# Supplementary material for: Experiences of Pacific Peoples and their ‘āiga/kāinga/kāiga/vuvale living with Parkinsons: A qualitative study
Source: PLoS One. 2026 Aug 12;21(8):e0355989. doi: 10.1371/journal.pone.0355989 (PMC13465971; doi:10.1371/journal.pone.0355989)
Supplement: Supplementary file 5 [file pone.0355989.s005.docx]

**PONE-D-26-01951R2: Author’s response to reviewer comments**

It is interesting that there is a parallel between the study group describing their experience as a four part journey equivalent to travelling in a traditional Polynesian outrigger canoe (va’a/vaka) and what we have come to identify as critical aspects to navigate in PD; 1) it’s onset is idiopathic or genetic (unexpected), 2) caregiver support is essential (who’s with me?), 3) getting educated about the best way to battle the disease (looking up at the stars to navigate), and looking for ways to beat it on a daily basis (steering the va’a/vaka).

Thank you for your review. We appreciate your considered comments. Addressing them has served to strength our paper. Our response to each comment is as follows and our changes in the paper are highlighted in yellow:

***1) Please define what Talanoa means at the first mention of it in the abstract.  I needed to look it up and found that Talanoa is supposed to represent inclusive, participatory and transparent dialogue-intended to build empathy, share stories, and fostering trust in the absence of a pretentious framework.  In my view, the Talanoa experience is similar to what a person with PD needs in caregiver support.***

Apologies for not defining Talanoa in the abstract. We have amended the abstract as follow (still remaining within our 300-word limit):

Talanoa research methodology [a Pacific phenomenological methodology founded on oral traditions of producing, sharing and transferring knowledge through conversation], eight consenting Pacific adults (>18 years) living in Aotearoa and diagnosed with Parkinson’s, irrespective of type, were invited to participate in talanoa [face-to-face conversations that understand the cultural relationality and connectedness of those involved].

***2) When it is stated on line 59 that Pacific peoples have the third highest prevalence of PD, please explain what third means; third as compared to which other groups?***

We have added the following to clarify:

… they have the third highest prevalence for Parkinson’s (160 per 100,000) of ethnic groups with known prevalence (namely, European, Asian, Māori), ….

***3) The reference to the biallelic p.L347P Pink1 variant of Pacific peoples in Aotearoa is a very compelling piece of information that may be quite pertinent to the age demographics of the 8 patients.  When subtracting the years since diagnosis from age of each study participant, the mean age of diagnosis was 40.9 years old.  This certainly qualifies as young onset, and the Pink1 mutation has been well-documented to produce young-onset PD, which 90% having onset before age 50.  The Pink1 mutation also shows a slower progression of the disease, which is also playing out in the demographics data wherein most of the patients in the study pool have a had PD longer than 10 years.  Please develop these aspects in the discussion.  Finally, are there genetic test results available on these study participants?***

This is a good point, and we have added to our discussion under clinical implications:

Continuation of health care services is especially important when working with Pacific Peoples. This important consideration is linked with our fourth key message that health professionals working with Pacific Peoples with Parkinson’s should be cognisant of the possibility of the PINK1 variant. Although we did not have access to generic profiling for those who may have been tested, consideration of our participant demographic data showed that many had early onset Parkinson’s. Six participants were under the age of 50, and one participant was 52 years of age, when their Parkinson’s signs and symptoms first appeared. Some participants commented that their healthcare professionals and the community support groups did not understand about their early onset and presentation. Whilst not much is currently known [61], the PINK1 variant appears characterised by an early-onset, a slow progression and lower limb dystonia, and thus its management may differ from those diagnosed with late-onset idiopathic disease [62]. This is crucial to understand given the long-term condition burden of those presenting with early onset Parkinson’s.

We have added “We had no access to genetic test results for any participants.” to the section in the Findings where we described participants’ demographic details.

***4) From the personal journey component, one critical observation shared by the group was their belief that physical activity was helpful (lines 473-483).  As we know that exercise is the only therapy that that attenuate the symptoms, it would be helpful to develop this section and determine more can be captured from the patient interviews.  Given that one of the premises of the paper is promoting awareness of the disease and to help others navigate this disease, it should be noted that the people in the group have identified, through their own journey, the necessity and helpfulness of physical activity.  Moreover, their practice of keeping physically active likely has contributed to their longevity in the battle. On this note, is it possible to get some reliable quantification of each individual’s accounting of their physical activity level; 1-2 times, 3-4 times, each day, per week and list that in table?***

We have added to the discussion a section on physical activity:

Our participants said they found engagement in physical exercise helpful. There is substantial evidence of the benefits of physical exercise on the severity of motor signs and quality of life of Parkinson’s [50], prompting the call for exercise to be considered as medicine in Parkinson’s and prescribed as an adjunct treatment as early as possible [51]. However, for Pacific Peoples, physical activity is deeply entwined with culture, community, spirituality, and identity, commingling everyday living, social connection, and cultural expression. Cultural values, gender roles, and the importance of social networks thus shape how people engage in physical activity [37,52]. An exploration of physical activity for Sāmoan stroke survivors challenged the value of the Western approach of medicalising physical activity, reducing it to a set of prescribed exercises of physical tasks, and proposed a holistic and relational approach integrating the spiritual, emotional, and social aspects of wellbeing [53]. This proposition could be extended to Pacific Peoples living with Parkinson’s.

We note your request regarding quantification of our participants’ physical activity, but we did not measure or capture such data (as this was not the aim of this qualitative study).
