# Supplementary material for: Experiences of Pacific Peoples and their ‘āiga/kāinga/kāiga/vuvale living with Parkinsons: A qualitative study
Source: PLoS One. 2026 Aug 12;21(8):e0355989. doi: 10.1371/journal.pone.0355989 (PMC13465971; doi:10.1371/journal.pone.0355989)
Supplement: Peer review history

# Peer review: Decision Letter 0

**DOI:** 10.1371/journal.pone.0355989.r001

**Authors:** Jen Edwards

23 Mar 2026

Dear Dr. Hale,

The manuscript has been evaluated by two reviewers, and their comments are available below. The reviewers have raised a number of major concerns regarding the presentation of the data, use of quotations and the discussion of clinical implications. Could you please carefully revise the manuscript to address all comments raised?

Please note that the reviewer comments include a recommendation to cite specific previously published works. Please review and evaluate these publications to determine whether they are relevant and should be cited; there is no requirement to cite these works.

Please submit your revised manuscript by May 04 2026 11:59PM. If you will need more time than this to complete your revisions, please reply to this message or contact the journal office at plosone@plos.org. When you're ready to submit your revision, log on to https://www.editorialmanager.com/pone/ and select the 'Submissions Needing Revision' folder to locate your manuscript file.

- A letter that responds to each point raised by the academic editor and reviewer(s). You should upload this letter as a separate file labeled 'Response to Reviewers'.
- A marked-up copy of your manuscript that highlights changes made to the original version. You should upload this as a separate file labeled 'Revised Manuscript with Track Changes'.
- An unmarked version of your revised paper without tracked changes. You should upload this as a separate file labeled 'Manuscript'.

If you would like to make changes to your financial disclosure, please include your updated statement in your cover letter. Guidelines for resubmitting your figure files are available below the reviewer comments at the end of this letter.

If applicable, we recommend that you deposit your laboratory protocols in protocols.io to enhance the reproducibility of your results. Protocols.io assigns your protocol its own identifier (DOI) so that it can be cited independently in the future. For instructions see: https://journals.plos.org/plosone/s/submission-guidelines#loc-laboratory-protocols. Additionally, PLOS ONE offers an option for publishing peer-reviewed Lab Protocol articles, which describe protocols hosted on protocols.io. Read more information on sharing protocols at https://plos.org/protocols?utm_medium=editorial-email&utm_source=authorletters&utm_campaign=protocols.

We look forward to receiving your revised manuscript.

Kind regards,

Jen Edwards

Staff Editor

PLOS One

Journal Requirements:

When submitting your revision, we need you to address these additional requirements.

1. Please ensure that your manuscript meets PLOS ONE's style requirements, including those for file naming. The PLOS ONE style templates can be found at

https://journals.plos.org/plosone/s/file?id=wjVg/PLOSOne_formatting_sample_main_body.pdf and https://journals.plos.org/plosone/s/file?id=ba62/PLOSOne_formatting_sample_title_authors_affiliations.pdf

2. Please include a complete copy of PLOS’ questionnaire on inclusivity in global research in your revised manuscript. Our policy for research in this area aims to improve transparency in the reporting of research performed outside of researchers’ own country or community. The policy applies to researchers who have travelled to a different country to conduct research, research with Indigenous populations or their lands, and research on cultural artefacts. The questionnaire can also be requested at the journal’s discretion for any other submissions, even if these conditions are not met.  Please find more information on the policy and a link to download a blank copy of the questionnaire here: https://journals.plos.org/plosone/s/best-practices-in-research-reporting. Please upload a completed version of your questionnaire as Supporting Information when you resubmit your manuscript.

3. For studies involving third-party data, we encourage authors to share any data specific to their analyses that they can legally distribute. PLOS recognizes, however, that authors may be using third-party data they do not have the rights to share. When third-party data cannot be publicly shared, authors must provide all information necessary for interested researchers to apply to gain access to the data. (https://journals.plos.org/plosone/s/data-availability#loc-acceptable-data-access-restrictions)

For any third-party data that the authors cannot legally distribute, they should include the following information in their Data Availability Statement upon submission:

1) A description of the data set and the third-party source

2) If applicable, verification of permission to use the data set

3) Confirmation of whether the authors received any special privileges in accessing the data that other researchers would not have

4) All necessary contact information others would need to apply to gain access to the data

4. We note that this manuscript consists of interview transcripts. Can you please confirm that all participants gave consent for interview transcript to be published?

If they DID provide consent for these transcripts to be published, please also confirm that the transcripts do not contain any potentially identifying information (or let us know if the participants consented to having their personal details published and made publicly available). We consider the following details to be identifying information:

- Names, nicknames, and initials

- Age more specific than round numbers

- GPS coordinates, physical addresses, IP addresses, email addresses

- Information in small sample sizes (e.g. 40 students from X class in X year at X university)

- Specific dates (e.g. visit dates, interview dates)

- ID numbers

Or, if the participants DID NOT provide consent for these transcripts to be published:

- Provide a de-identified version of the data or excerpts of interview responses

- Provide information regarding how these transcripts can be accessed by researchers who meet the criteria for access to confidential data, including:

a) the grounds for restriction

b) the name of the ethics committee, Institutional Review Board, or third-party organization that is imposing sharing restrictions on the data

c) a non-author, institutional point of contact that is able to field data access queries, in the interest of maintaining long-term data accessibility.

d) Any relevant data set names, URLs, DOIs, etc. that an independent researcher would need in order to request your minimal data set.

For further information on sharing data that contains sensitive participant information, please see: https://journals.plos.org/plosone/s/data-availability#loc-human-research-participant-data-and-other-sensitive-data

If there are ethical, legal, or third-party restrictions upon your dataset, you must provide all of the following details (https://journals.plos.org/plosone/s/data-availability#loc-acceptable-data-access-restrictions):

1. A complete description of the dataset

2. The nature of the restrictions upon the data (ethical, legal, or owned by a third party) and the reasoning behind them

3. The full name of the body imposing the restrictions upon your dataset (ethics committee, institution, data access committee, etc)

4. If the data are owned by a third party, confirmation of whether the authors received any special privileges in accessing the data that other researchers would not have

5. Direct, non-author contact information (preferably email) for the body imposing the restrictions upon the data, to which data access requests can be sent.

5. Please include captions for your Supporting Information files at the end of your manuscript, and update any in-text citations to match accordingly. Please see our Supporting Information guidelines for more information: http://journals.plos.org/plosone/s/supporting-information.

If the reviewer comments include a recommendation to cite specific previously published works, please review and evaluate these publications to determine whether they are relevant and should be cited. There is no requirement to cite these works unless the editor has indicated otherwise.

[Note: HTML markup is below. Please do not edit.]

Reviewers' comments:

Reviewer's Responses to Questions

**Comments to the Author**

1. Is the manuscript technically sound, and do the data support the conclusions?

Reviewer #1: Yes

Reviewer #2: Yes

**********

2. Has the statistical analysis been performed appropriately and rigorously?

Reviewer #1: N/A

Reviewer #2: Yes

**********

3. Have the authors made all data underlying the findings in their manuscript fully available?

The PLOS Data policy

Reviewer #1: No

Reviewer #2: No

**********

4. Is the manuscript presented in an intelligible fashion and written in standard English?

Reviewer #1: Yes

Reviewer #2: Yes

**********

Reviewer #1: This manuscript is an important contribution to the PD community to enhance diversity in the representation of PD voices. It is a solid step towards understanding the issues faced by the Pacific People PD patients, and hopefully improve the awareness and support for the patients and their families. I am sure there was a lot of data collected during the interviews which was difficult to distill for this manuscript, however the manuscript would benefit from additional information to help the readers understand the quotes and findings more clearly.

Major point

1) I could not find a detailed demographic table of the participants, only the generalised summary of their ages and conditions. It would be good to include this, without giving away too much personal identifiable detail.

2) There were 8 interviewees but I could only find quotes for 5 (P1, P18, P21, P22, P23 and P24). Could the authors include quotes from the other interviewees as well? It seemed as if the majority of the quotes stated come from P18 and P22, it would be good if a more equal distribution was given to the others.

3) The questions in the interview guide were very insightful, but I was not able to see where the answers for some of the important questions like Did you seek medical advice and what was you experience with seeking medical advice? (except for one quote by P22) as this could have direct impact on how the local medical community could be better educated when discussing the PD diagnosis with the patients and their families.

4) The statement 'Advocacy from family was important' could be expanded further.

5) If possible, it would be good to add more quotes of positive perspectives, especially for the section "Steering your va’a/vaka – finding your way", this would be also a nice way to highlight the resilience of the participants and begin to tease out some of the meaningful and innovative changes/mindset they have developed as their lived experience is a precious resource for others to learn from.

Minor comments

1) The word Parkinson's should be referred to as Parkinson's disease or PD in the intro/methods etc as this is its proper term, but can be kept as is if this is the term that was used by the participants' quotes.

2) It is good that the authors relayed their findings to the community as an engagement exercise, it is not clear whether there is an local association/society/regional chapters through which more activities can be initiated. They may want to include this as a means to highlight the work they do.

Reviewer #2: This manuscript presents a qualitative exploration of the experiences of Pacific Peoples living with Parkinson’s disease in Aotearoa New Zealand using Talanoa methodology and reflexive thematic analysis. The study addresses an important and under-researched population and provides culturally grounded insights into the lived experiences of Parkinson’s disease among Pacific communities.

Overall, the manuscript is technically sound, and the qualitative design is appropriate for the research question. The use of the Talanoa methodology and reflexive thematic analysis is well justified, and the involvement of Pacific researchers strengthens the cultural validity of the research approach. The themes identified are coherent and supported by illustrative participant quotations. The study contributes valuable insights into culturally informed care and the social context of Parkinson’s disease.

However, several issues should be addressed to strengthen the manuscript.

1.The study includes eight participants. While this sample size is acceptable for qualitative research, the authors should clarify how they assessed thematic sufficiency or saturation. A short explanation regarding how the research team determined that the dataset was adequate for thematic development would strengthen methodological transparency.

2.Although the study is primarily qualitative, several clinical assessments were collected (e.g., MDS-UPDRS III, TUG, fall risk indicators). The rationale for including these quantitative measures should be clarified. At present, they are only briefly summarized and do not clearly convey the qualitative findings. The authors may either better explain their contextual purpose or consider relocating them to supplementary information.

3.The discussion on social isolation could be strengthened by incorporating evidence on functional outcomes in PD. Social isolation and reduced community participation were evident in participants’ narratives. Social functioning is increasingly recognized as an important outcome in PD, as motor symptoms, cognitive changes, and stigma may limit engagement in social roles and daily activities (Ahn et al., 2022; Su et al., 2020; Yu et al., 2022). Refs: Ahn et al. Geriatric Nursing 48 (2022): 258-268. Su et al. Journal of Parkinson’s Disease 10.3 (2020): 1143-1151.;Yu et al. Frontiers in aging neuroscience 14 (2022): 913958.

4.The discussion appropriately links findings to existing qualitative literature on Parkinson’s disease and stigma. However, the authors may strengthen the manuscript by More clearly articulating the clinical implications for healthcare services. In addition, highlighting how culturally responsive care models could be implemented in practice. Moreover, clarifying how the proposed community resources (videos, educational materials, etc.) will be translated into health service improvements.

**********

what does this mean?). If published, this will include your full peer review and any attached files.

If you choose “no”, your identity will remain anonymous but your review may still be made public.

**Do you want your identity to be public for this peer review?**  For information about this choice, including consent withdrawal, please see our Privacy Policy

Reviewer #1: No

Reviewer #2: No

**********

[NOTE: If reviewer comments were submitted as an attachment file, they will be attached to this email and accessible via the submission site. Please log into your account, locate the manuscript record, and check for the action link "View Attachments". If this link does not appear, there are no attachment files.]

To ensure your figures meet our technical requirements, please review our figure guidelines: https://journals.plos.org/plosone/s/figures

You may also use PLOS’s free figure tool, NAAS, to help you prepare publication quality figures: https://journals.plos.org/plosone/s/figures#loc-tools-for-figure-preparation.

NAAS will assess whether your figures meet our technical requirements by comparing each figure against our figure specifications.

# Author comment: Author response to Decision Letter 1

**DOI:** 10.1371/journal.pone.0355989.r002

30 Mar 2026

We have uploaded an Authors Response to reviewers addressing all comments and request from the Editor and the reviewers in the decision letter.

Attachment Submitted filename: Authors response to reviewers PONE-D-26-01951.docx

# Peer review: Decision Letter 1

**DOI:** 10.1371/journal.pone.0355989.r003

**Authors:** Katrien Janin

14 May 2026

Dear Dr. Hale,

Thank you for submitting your manuscript to PLOS ONE. After careful consideration, we feel that it has merit but does not fully meet PLOS ONE’s publication criteria as it currently stands. Therefore, we invite you to submit a revised version of the manuscript that addresses the points raised during the review process.

Your manuscript has now been reassessed. While the reviewer acknowledges the improvements made to the manuscript, they have also highlighted the remaining issues that should be addressed - mainly the methodological rigor needs to be strengthen. Please take these points into careful consideration when preparing your revised manuscript and your point‑by‑point response.

Please submit your revised manuscript by Jun 28 2026 11:59PM. If you will need more time than this to complete your revisions, please reply to this message or contact the journal office at plosone@plos.org. When you're ready to submit your revision, log on to https://www.editorialmanager.com/pone/ and select the 'Submissions Needing Revision' folder to locate your manuscript file.

- A letter that responds to each point raised by the academic editor and reviewer(s). You should upload this letter as a separate file labeled 'Response to Reviewers'.
- A marked-up copy of your manuscript that highlights changes made to the original version. You should upload this as a separate file labeled 'Revised Manuscript with Track Changes'.
- An unmarked version of your revised paper without tracked changes. You should upload this as a separate file labeled 'Manuscript'.

If you would like to make changes to your financial disclosure, please include your updated statement in your cover letter. Guidelines for resubmitting your figure files are available below the reviewer comments at the end of this letter.

If applicable, we recommend that you deposit your laboratory protocols in protocols.io to enhance the reproducibility of your results. Protocols.io assigns your protocol its own identifier (DOI) so that it can be cited independently in the future. For instructions see: https://journals.plos.org/plosone/s/submission-guidelines#loc-laboratory-protocols. Additionally, PLOS ONE offers an option for publishing peer-reviewed Lab Protocol articles, which describe protocols hosted on protocols.io. Read more information on sharing protocols at https://plos.org/protocols?utm_medium=editorial-email&utm_source=authorletters&utm_campaign=protocols.

As the corresponding author, your ORCID iD is verified in the submission system and will appear in the published article. PLOS supports the use of ORCID, and we encourage all coauthors to register for an ORCID iD and use it as well. Please encourage your coauthors to verify their ORCID iD within the submission system before final acceptance, as unverified ORCID iDs will not appear in the published article. *Only*  the individual author can complete the verification step; PLOS staff *cannot*  verify ORCID iDs on behalf of authors.

We look forward to receiving your revised manuscript.

Kind regards,

Katrien G. Janin, PhD

Staff Editor

PLOS One

Journal Requirements:

If the reviewer comments include a recommendation to cite specific previously published works, please review and evaluate these publications to determine whether they are relevant and should be cited. There is no requirement to cite these works unless the editor has indicated otherwise.

[Note: HTML markup is below. Please do not edit.]

Reviewers' comments:

Reviewer's Responses to Questions

**Comments to the Author**

Reviewer #1: All comments have been addressed

Reviewer #2: (No Response)

**********

2. Is the manuscript technically sound, and do the data support the conclusions?

Reviewer #1: Yes

Reviewer #2: No

**********

3. Has the statistical analysis been performed appropriately and rigorously?

Reviewer #1: N/A

Reviewer #2: No

**********

4. Have the authors made all data underlying the findings in their manuscript fully available?

The PLOS Data policy

Reviewer #1: Yes

Reviewer #2: No

**********

5. Is the manuscript presented in an intelligible fashion and written in standard English?

Reviewer #1: Yes

Reviewer #2: No

**********

Reviewer #1: Regarding the response:

"As is usual in qualitative research, some participants were verbose and some did not say very much

(P15 and P17 fall into this latter category)". It is still important to include quotes from all participants in qualitative papers, and especially since the thematic analysis suggests that they would have all said something that would have reflected that specific point, there should not have been an issue selecting quotes from all the participants.

Reviewer #2: Thanks for the revision. The authors answered each question briefly; however, the major concerns have not been meaningfully resolved. The study still relies on a very small and clinically heterogeneous sample, without adequately justifying how such limited data can support the current thematic structure. In addition, the clinical composition of the sample remains insufficiently defined, the description of the analysis is still not fully consistent with reflexive thematic analysis, the boundary between primary interview data and later community “verification” remains unclear, and the handling of multilingual data is still underexplained. Overall, although the revision improves presentation, it does not materially strengthen the manuscript’s methodological rigor.

**********

what does this mean?). If published, this will include your full peer review and any attached files.

If you choose “no”, your identity will remain anonymous but your review may still be made public.

**Do you want your identity to be public for this peer review?**  For information about this choice, including consent withdrawal, please see our Privacy Policy

Reviewer #1: No

Reviewer #2: No

**********

[NOTE: If reviewer comments were submitted as an attachment file, they will be attached to this email and accessible via the submission site. Please log into your account, locate the manuscript record, and check for the action link "View Attachments". If this link does not appear, there are no attachment files.]

To ensure your figures meet our technical requirements, please review our figure guidelines: https://journals.plos.org/plosone/s/figures

You may also use PLOS’s free figure tool, NAAS, to help you prepare publication quality figures: https://journals.plos.org/plosone/s/figures#loc-tools-for-figure-preparation.

NAAS will assess whether your figures meet our technical requirements by comparing each figure against our figure specifications.

# Author comment: Author response to Decision Letter 2

**DOI:** 10.1371/journal.pone.0355989.r004

24 May 2026

PONE-D-26-01951 Authors Response to Reviewers' Comments

Thank you to the reviewers who have taken time and consideration to review our revised version of our paper. This has enabled us to strengthen our paper further. Below we respond to each comment and in our revised paper we have highlighted the changes made in yellow.

Reviewer #1: Regarding the response:

"As is usual in qualitative research, some participants were verbose and some did not say very much (P15 and P17 fall into this latter category)". It is still important to include quotes from all participants in qualitative papers, and especially since the thematic analysis suggests that they would have all said something that would have reflected that specific point, there should not have been an issue selecting quotes from all the participants.

Authors’ Response: Please note that in our first revised manuscript we did have quotes from all participants, but we have now added more quotes from P15 and P17.

Reviewer #2: Thanks for the revision. The authors answered each question briefly; however, the major concerns have not been meaningfully resolved.

Authors’ Response: We are sorry if our previsions revisions were a little sparse. We have now revised further by expanding our explanations in the manuscript to each of your points to provide greater clarity (see yellow highlighted text).

Methodological approach: Under ‘Design’: see lines 119-128.

Reviewer #2: The study still relies on a very small and clinically heterogeneous sample, without adequately justifying how such limited data can support the current thematic structure.

Authors’ Response: Under ‘Participants and Recruitment’: see lines 157-160 and ‘Strength and Limitations’: see lines 707-717.

Reviewer #2: In addition, the clinical composition of the sample remains insufficiently defined,

Authors’ Response: We are not sure what more we can add here – we have clinically defined our sample (see lines 260-285, Table 1) and we have discussed under ‘Strengths and limitations’ (lines 713-717). Given that we had 8 participants, many who came from the same area, we could not be more specific in our reporting (such as a more detailed participant demographic table) without identifying our participants and thereby contravening our ethical approval.

Reviewer #2: The description of the analysis is still not fully consistent with reflexive thematic analysis,

Authors’ Response: Under ‘Data analysis’: see lines197-228.

Reviewer #2: The boundary between primary interview data and later community “verification” remains unclear,

Authors’ Response: Under ‘Second stage community and participants consultation’: see lines 220-228

Reviewer #2: The handling of multilingual data is still underexplained.

Authors’ Response: Under ‘Data collection’ lines186-188 and then Under ‘Data analysis’: see lines 197-203.

Reviewer #2: Overall, although the revision improves presentation, it does not materially strengthen the manuscript’s methodological rigor.

Authors’ Response: We hope that we have now strengthened our manuscript’s methodological rigour, underpinned by our non-positivist ‘Big Q’ Qualitative research approach as opposed to a post-positivist “q” approach as referenced. (Kidder LH, Fine M. 1987. Qualitative and quantitative methods: When stories converge. In M. M. Mark & L. Shotland (Eds.), New directions for program evaluation (pp. 57–75). Jossey-Bass. doi: 10.1002/ev.1459; Braun V, Clarke V. Supporting best practice in reflexive thematic analysis reporting in Palliative Medicine: A review of published research and introduction to the Reflexive Thematic Analysis Reporting Guidelines (RTARG). Palliative Med., 2024;38(6):608-616; doi: 10.1177/02692163241234800; Braun V, Clarke V. 2013. Successful qualitative research: A practical guide for beginners. Sage.)

Attachment Submitted filename: Response to Reviewers comments _R2.docx

# Peer review: Decision Letter 2

**DOI:** 10.1371/journal.pone.0355989.r005

**Authors:** Michael Salvatore

21 Jul 2026

Dear Dr. Hale,

Thank you for submitting your manuscript to PLOS ONE. After careful consideration, we feel that it has merit but does not fully meet PLOS ONE’s publication criteria as it currently stands. Therefore, we invite you to submit a revised version of the manuscript that addresses the points raised during the review process.

==============================

Notes from the editor:

It is interesting that there is a parallel between the study group describing their experience as a four part journey equivalent to travelling in a traditional Polynesian outrigger canoe (va’a/vaka) and what we have come to identify as critical aspects to navigate in PD; 1) it’s onset is idiopathic or genetic (unexpected), 2) caregiver support is essential (who’s with me?), 3) getting educated about the best way to battle the disease (looking up at the stars to navigate), and looking for ways to beat it on a daily basis (steering the va’a/vaka).

1) Please define what Talanoa means at the first mention of it in the abstract.  I needed to look it up and found that Talanoa is supposed to represent inclusive, participatory and transparent dialogue-intended to build empathy, share stories, and fostering trust in the absence of a pretentious framework.  In my view,  the Talanoa experience is similar to what a person with PD needs in caregiver support.

2) When it is stated on line 59 that Pacific peoples have the third highest prevalence of PD, please explain what third means; third as compared to which other groups?

3) The reference to the biallelic p.L347P Pink1 variant of Pacific peoples in Aotearoa is a very compelling piece of information that may be quite pertinent to the age demographics of the 8 patients.  When subtracting the years since diagnosis from age of each study participant, the mean age of diagnosis was 40.9 years old.  This certainly qualifies as young onset, and the Pink1 mutation has been well-documented to produce young-onset PD, which 90% having onset before age 50.  The Pink1 mutation also shows a slower progression of the disease, which is also playing out in the demographics data wherein most of the patients in the study pool have a had PD longer than 10 years.  Please develop these aspects in the discussion.  Finally, are there genetic test results available on these study participants?

4) From the personal journey component, one critical observation shared by the group was their belief that physical activity was helpful (lines 473-483).  As we know that exercise is the only therapy that that attenuate the symptoms, it would be helpful to develop this section and determine more can be captured from the patient interviews.  Given that one of the premises of the paper is promoting awareness of the disease and to help others navigate this disease, it should be noted that the people in the group have identified, through their own journey, the necessity and helpfulness of physical activity.  Moreover, their practice of keeping physically active likely has contributed to their longevity in the battle. On this note, is it possible to get some reliable quantification of each individual’s accounting of their physical activity level; 1-2 times, 3-4 times, each day, per week and list that in table?

==============================

Please submit your revised manuscript by Sep 04 2026 11:59PM. If you will need more time than this to complete your revisions, please reply to this message or contact the journal office at plosone@plos.org. When you're ready to submit your revision, log on to https://www.editorialmanager.com/pone/ and select the 'Submissions Needing Revision' folder to locate your manuscript file.

- A letter that responds to each point raised by the academic editor and reviewer(s). You should upload this letter as a separate file labeled 'Response to Reviewers'.
- A marked-up copy of your manuscript that highlights changes made to the original version. You should upload this as a separate file labeled 'Revised Manuscript with Track Changes'.
- An unmarked version of your revised paper without tracked changes. You should upload this as a separate file labeled 'Manuscript'.

If you would like to make changes to your financial disclosure, please include your updated statement in your cover letter. Guidelines for resubmitting your figure files are available below the reviewer comments at the end of this letter.

If applicable, we recommend that you deposit your laboratory protocols in protocols.io to enhance the reproducibility of your results. Protocols.io assigns your protocol its own identifier (DOI) so that it can be cited independently in the future. For instructions see: https://journals.plos.org/plosone/s/submission-guidelines#loc-laboratory-protocols. Additionally, PLOS ONE offers an option for publishing peer-reviewed Lab Protocol articles, which describe protocols hosted on protocols.io. Read more information on sharing protocols at https://plos.org/protocols?utm_medium=editorial-email&utm_source=authorletters&utm_campaign=protocols.

As the corresponding author, your ORCID iD is verified in the submission system and will appear in the published article. PLOS supports the use of ORCID, and we encourage all coauthors to register for an ORCID iD and use it as well. Please encourage your coauthors to verify their ORCID iD within the submission system before final acceptance, as unverified ORCID iDs will not appear in the published article. *Only*  the individual author can complete the verification step; PLOS staff *cannot*  verify ORCID iDs on behalf of authors.

We look forward to receiving your revised manuscript.

Kind regards,

Michael Francis Salvatore

Academic Editor

PLOS One

Journal Requirements:

If the reviewer comments include a recommendation to cite specific previously published works, please review and evaluate these publications to determine whether they are relevant and should be cited. There is no requirement to cite these works unless the editor has indicated otherwise.

Please review your reference list to ensure that it is complete and correct. If you have cited papers that have been retracted, please include the rationale for doing so in the manuscript text, or remove these references and replace them with relevant current references. Any changes to the reference list should be mentioned in the rebuttal letter that accompanies your revised manuscript. If you need to cite a retracted article, indicate the article’s retracted status in the References list and also include a citation and full reference for the retraction notice.

Additional Editor Comments:

It is interesting that there is a parallel between the study group describing their experience as a four part journey equivalent to travelling in a traditional Polynesian outrigger canoe (va’a/vaka) and what we have come to identify as critical aspects to navigate in PD; 1) it’s onset is idiopathic or genetic (unexpected), 2) caregiver support is essential (who’s with me?), 3) getting educated about the best way to battle the disease (looking up at the stars to navigate), and looking for ways to beat it on a daily basis (steering the va’a/vaka).

1) Please define what Talanoa means at the first mention of it in the abstract. I needed to look it up and found that Talanoa is supposed to represent inclusive, participatory and transparent dialogue-intended to build empathy, share stories, and fostering trust in the absence of a pretentious framework. In my view, the Talanoa experience is similar to what a person with PD needs in caregiver support.

2) When it is stated on line 59 that Pacific peoples have the third highest prevalence of PD, please explain what third means; third as compared to which other groups?

3) The reference to the biallelic p.L347P Pink1 variant of Pacific peoples in Aotearoa is a very compelling piece of information that may be quite pertinent to the age demographics of the 8 patients. When subtracting the years since diagnosis from age of each study participant, the mean age of diagnosis was 40.9 years old. This certainly qualifies as young onset, and the Pink1 mutation has been well-documented to produce young-onset PD, which 90% having onset before age 50. The Pink1 mutation also shows a slower progression of the disease, which is also playing out in the demographics data wherein most of the patients in the study pool have a had PD longer than 10 years. Please develop these aspects in the discussion. Finally, are there genetic test results available on these study participants?

4) From the personal journey component, one critical observation shared by the group was their belief that physical activity was helpful (lines 473-483). As we know that exercise is the only therapy that that attenuate the symptoms, it would be helpful to develop this section and determine more can be captured from the patient interviews. Given that one of the premises of the paper is promoting awareness of the disease and to help others navigate this disease, it should be noted that the people in the group have identified, through their own journey, the necessity and helpfulness of physical activity. Moreover, their practice of keeping physically active likely has contributed to their longevity in the battle. On this note, is it possible to get some reliable quantification of each individual’s accounting of their physical activity level; 1-2 times, 3-4 times, each day, per week and list that in table?

[Note: HTML markup is below. Please do not edit.]

Reviewers' comments:

Reviewer's Responses to Questions

**Comments to the Author**

Reviewer #1: All comments have been addressed

**********

2. Is the manuscript technically sound, and do the data support the conclusions?

Reviewer #1: Yes

**********

3. Has the statistical analysis been performed appropriately and rigorously?

Reviewer #1: N/A

**********

4. Have the authors made all data underlying the findings in their manuscript fully available?

The PLOS Data policy

Reviewer #1: Yes

**********

5. Is the manuscript presented in an intelligible fashion and written in standard English?

Reviewer #1: Yes

**********

Reviewer #1: (No Response)

**********

what does this mean?). If published, this will include your full peer review and any attached files.

If you choose “no”, your identity will remain anonymous but your review may still be made public.

**Do you want your identity to be public for this peer review?**  For information about this choice, including consent withdrawal, please see our Privacy Policy

Reviewer #1: No

**********

[NOTE: If reviewer comments were submitted as an attachment file, they will be attached to this email and accessible via the submission site. Please log into your account, locate the manuscript record, and check for the action link "View Attachments". If this link does not appear, there are no attachment files.]

To ensure your figures meet our technical requirements, please review our figure guidelines: https://journals.plos.org/plosone/s/figures

You may also use PLOS’s free figure tool, NAAS, to help you prepare publication quality figures: https://journals.plos.org/plosone/s/figures#loc-tools-for-figure-preparation.

NAAS will assess whether your figures meet our technical requirements by comparing each figure against our figure specifications.

# Author comment: Author response to Decision Letter 3

**DOI:** 10.1371/journal.pone.0355989.r006

27 Jul 2026

We have uploaded a separate file detailing our response to the reviewers' comments

Attachment Submitted filename: Response to Reviewers.docx

# Editor report: Decision Letter 3

**DOI:** 10.1371/journal.pone.0355989.r007

**Authors:** Michael Salvatore

28 Jul 2026

Experiences of Pacific Peoples and their ‘āiga/kāinga/kāiga/vuvale living with Parkinsons: A qualitative study

PONE-D-26-01951R3

Dear Dr. Hale,

We’re pleased to inform you that your manuscript has been judged scientifically suitable for publication and will be formally accepted for publication once it meets all outstanding technical requirements.

Within one week, you’ll receive an e-mail detailing the required amendments. When these have been addressed, you’ll receive a formal acceptance letter and your manuscript will be scheduled for publication.

An invoice will be generated when your article is formally accepted. Please note, if your institution has a publishing partnership with PLOS and your article meets the relevant criteria, all or part of your publication costs will be covered. Please make sure your user information is up-to-date by logging into Editorial Manager at Editorial Manager® and clicking the ‘Update My Information' link at the top of the page. For questions related to billing, please contact billing support.

If your institution or institutions have a press office, please notify them about your upcoming paper to help maximize its impact. If they’ll be preparing press materials, please inform our press team as soon as possible -- no later than 48 hours after receiving the formal acceptance. Your manuscript will remain under strict press embargo until 2 pm Eastern Time on the date of publication. For more information, please contact onepress@plos.org.

Kind regards,

Michael Francis Salvatore

Academic Editor

PLOS One

Additional Editor Comments (optional):

Reviewers' comments:

# Editor report: Acceptance letter

**DOI:** 10.1371/journal.pone.0355989.r008

**Authors:** Michael Salvatore

PONE-D-26-01951R3

PLOS One

Dear Dr. Hale,

I'm pleased to inform you that your manuscript has been deemed suitable for publication in PLOS One. Congratulations! Your manuscript is now being handed over to our production team.

At this stage, our production department will prepare your paper for publication. This includes ensuring the following:

* All references, tables, and figures are properly cited

* All relevant supporting information is included in the manuscript submission,

* There are no issues that prevent the paper from being properly typeset

You will receive further instructions from the production team, including instructions on how to review your proof when it is ready. Please keep in mind that we are working through a large volume of accepted articles, so please give us a few days to review your paper and let you know the next and final steps.

Lastly, if your institution or institutions have a press office, please let them know about your upcoming paper now to help maximize its impact. If they'll be preparing press materials, please inform our press team within the next 48 hours. Your manuscript will remain under strict press embargo until 2 pm Eastern Time on the date of publication. For more information, please contact onepress@plos.org.

You will receive an invoice from PLOS for your publication fee after your manuscript has reached the completed accept phase. If you receive an email requesting payment before acceptance or for any other service, this may be a phishing scheme. Learn how to identify phishing emails and protect your accounts at https://explore.plos.org/phishing.

If we can help with anything else, please email us at customercare@plos.org.

Thank you for submitting your work to PLOS ONE and supporting open access.

Kind regards,

PLOS ONE Editorial Office Staff

on behalf of

Dr. Michael Francis Salvatore

Academic Editor

PLOS One
